# Extracellular vesicles derived from bone marrow mesenchymal stem cells regulate SREBF2/HMGB1 axis by transporting miR-378a-3p to inhibit ferroptosis in intestinal ischemia-reperfusion injury

**DOI:** 10.1038/s41420-025-02509-6

**Published:** 2025-05-07

**Authors:** Zan Liu, Zitong Zhao, Zhenghui Xiao, Ming Li, Xiyang Wang, Yan Huang, Yong Li

**Affiliations:** 1 Department of Pediatric Surgery, Clinical Research Center for Pediatric Solid Tumors in Hunan Province, Hunan Provincial Key Laboratory of Pediatric Orthopedics, The Affiliated Children’s Hospital of Xiangya School of Medicine, Central South University (Hunan children’s hospital), Changsha, PR China; 2 Hunan Provincial Key Laboratory of the Research and Development of Novel Pharmaceutical Preparations, College of Pharmacy, Changsha Medical University, Changsha, PR China; 3 Center of Reproductive Medicine, Changsha Hospital for Maternal and Child Health Care of Hunan Normal University, Changsha, PR China; 4 Emergency center of Hunan Children’s Hospital, Changsha, Hunan PR China; 5 Department of Spine Surgery, Xiangya Hospital, Central South University, Changsha, Hunan PR China; 6 NHC Key Laboratory of Birth Defect for Research and Prevention, Hunan Provincial Maternal and Child Health Care Hospital, Changsha, Hunan PR China; 7 Hunan Provincial Key Laboratory of Neurorestoration, Changsha, Hunan PR China

**Keywords:** Necroptosis, Anaemia, Mesenchymal stem cells, Experimental models of disease

## Abstract

Intestinal ischemia-reperfusion (II/R) injury represents a life-threatening and complex pathophysiological process that remains challenging to treat clinically, and emerging evidence suggests that ferroptosis plays an essential role in its pathogenesis. This study aimed to investigate whether extracellular vesicles derived from bone marrow mesenchymal stem cells (BMSC-EVs) can mitigate II/R-induced ferroptosis in a murine model. Using a bioinformatics database, we initially identified genes with abnormal expression patterns in II/R injury. Then, we confirmed the association between II/R injury, ferroptosis, and the HMGB1/SREBF2 axis through in vivo and in vitro experiments. To determine the role of HMGB1 in hypoxia/reoxygenation (H/R)-induced ferroptosis in Caco-2 cells, we transfected cells with either sh-HMGB1 or control sh-NC constructs and developed an H/R model in vitro. Subsequently, we examined factors regulating HMGB1-mediated ferroptosis in Caco-2 cells and assessed the effect of BMSC-EVs on this process. To further explore the mechanism underlying the protective effects of BMSC-EVs in II/R injury, we screened for miRNAs with reduced expression during II/R and verified their involvement. Among these, miR-378a-3p was identified as a candidate for regulating ferroptosis. To confirm its functional role, we treated II/R mice with BMSC-EVs overexpressing miR-378a-3p and assessed the outcomes. Our findings revealed that HMGB1, which is a key regulatory factor of ferroptosis, was significantly upregulated during II/R injury, and its knockdown alleviated H/R-induced ferroptosis in Caco-2 cells. We also found that SREBF2 directly regulates HMGB1 expression to promote H/R-induced ferroptosis in vitro. Importantly, BMSC-EVs alleviated II/R injury by suppressing ferroptosis in Caco-2 cells, and mechanistically, miR-378a-3p, a miRNA derived from BMSC-EVs, inhibited II/R-induced ferroptosis by modulating the SREBF2/HMGB1 axis. In conclusion, BMSC-EVs may exert protective effects against II/R injury by delivering miR-378a-3p, which regulates the SREBF2/HMGB1 axis to suppress ferroptosis, providing important insights into the pathological mechanisms underlying II/R injury and potential therapeutic strategies for its management.

## Introduction

Intestinal ischemia-reperfusion (II/R) injury is a critical vascular emergency often associated with localized medical procedures such as acute mesenteric ischemia, hemorrhagic or septic shock, severe burns, and specific surgical interventions, including cardiopulmonary bypass, small-bowel transplantation, and abdominal aortic surgery [1–3]. It can lead to localized intestinal damage, where extensive epithelial cell death disrupts the intestinal mucosal barrier, resulting in systemic inflammation and dysfunction of distant organs such as the lungs, liver, kidneys, heart, and brain, leading to potentially fatal outcomes [4]. Despite its clinical significance, effective treatment of II/R injuries remains a considerable challenge due to an incomplete understanding of its underlying mechanisms and the lack of effective therapeutic options.

Recent studies have highlighted the promising role of ferroptosis as a pivotal mechanism contributing to cellular and organ damage during I/R events [5]. Ferroptosis is characterized by mitochondrial constriction, increased mitochondrial membrane density and the accumulation of iron and lipid reactive oxygen species (ROS), distinct from other forms of programmed cell death such as apoptosis, necrosis, and pyroptosis [6, 7]. The primary trigger of ferroptosis is the dysregulation of lipid ROS production and clearance within cells. Biochemically, ferroptosis is driven by iron-catalyzed consumption of lipid radicals, depletion of glutathione (GSH), and inactivation of glutathione peroxidase 4 (GPX4). These processes amplify lipid peroxidation through the Fenton reaction, leading to ROS accumulation and, ultimately, cell death [8, 9]. While non-apoptotic pathways such as ferroptosis are increasingly recognized as significant contributors to II/R injury [10, 11], the precise mechanisms underlying ferroptosis induction in intestinal tissues during II/R injury remain poorly understood.

Bone marrow mesenchymal stem cells (BMSCs) are currently among the most extensively studied types of stem cells and have gained prominence due to their significant advancements in regenerative medicine and tissue engineering. BMSCs are recognized for their therapeutic potential in treating various diseases, including bone healing, autoimmune disorders, and inflammatory conditions. Their capacity to differentiate into multiple cell types and secrete bioactive molecules is essential in regenerative therapies [12]. While BMSC treatment has been shown to reduce intestinal injury caused by II/R, the precise mechanisms underlying this effect remain unclear [13, 14]. Thus, while BMSCs are versatile and have promising clinical potential, there is a continuous need for further research to optimize their application in regenerative medicine. BMSCs primarily repair damaged tissues through strong paracrine effects, with extracellular vesicles (EVs), including exosomes, playing a pivotal role. EVs, secreted by nearly all cell types, facilitate intercellular communication by transporting bioactive molecules such as mRNAs, non-coding RNAs, lipids, and proteins [15]. Intracellular delivery of EVs has been demonstrated in various cell types, enabling the functional utilization of delivered miRNAs [16]. Recent studies have shown that BMSC-derived exosomes exhibit therapeutic effects in models of myocardial infarction [17] and I/R injury in steatotic grafts [18]. However, the specific roles and mechanisms by which BMSC-EVs regulate II/R-induced ferroptosis remain largely unexplored.

In this study, we investigated the potential of BMSC-EVs in mitigating II/R-induced ferroptosis in a murine model and explored the significance of the miR-378a-3p/sterol regulatory element -binding transcription factor 2 (SREBF2)/high mobility group box 1 (HMGB1) axis in mediating the suppression of II/R- and hypoxia/reoxygenation (H/R)-induced ferroptosis by BMSC-EVs, both in vivo and in vitro. Our findings demonstrated that BMSC-EVs exert their protective effects by modulating the SREBF2/HMGB1 axis through the delivery of miR-378a-3p, thereby inhibiting ferroptosis in II/R-affected cells, suggesting that miR-378a-3p delivered via BMSC-EVs represents a promising therapeutic candidate for the treatment of II/R-induced ferroptosis.

## Results

### Lip or HMGB1 inhibited ferroptosis induced by II/R

**Fig. 1:**
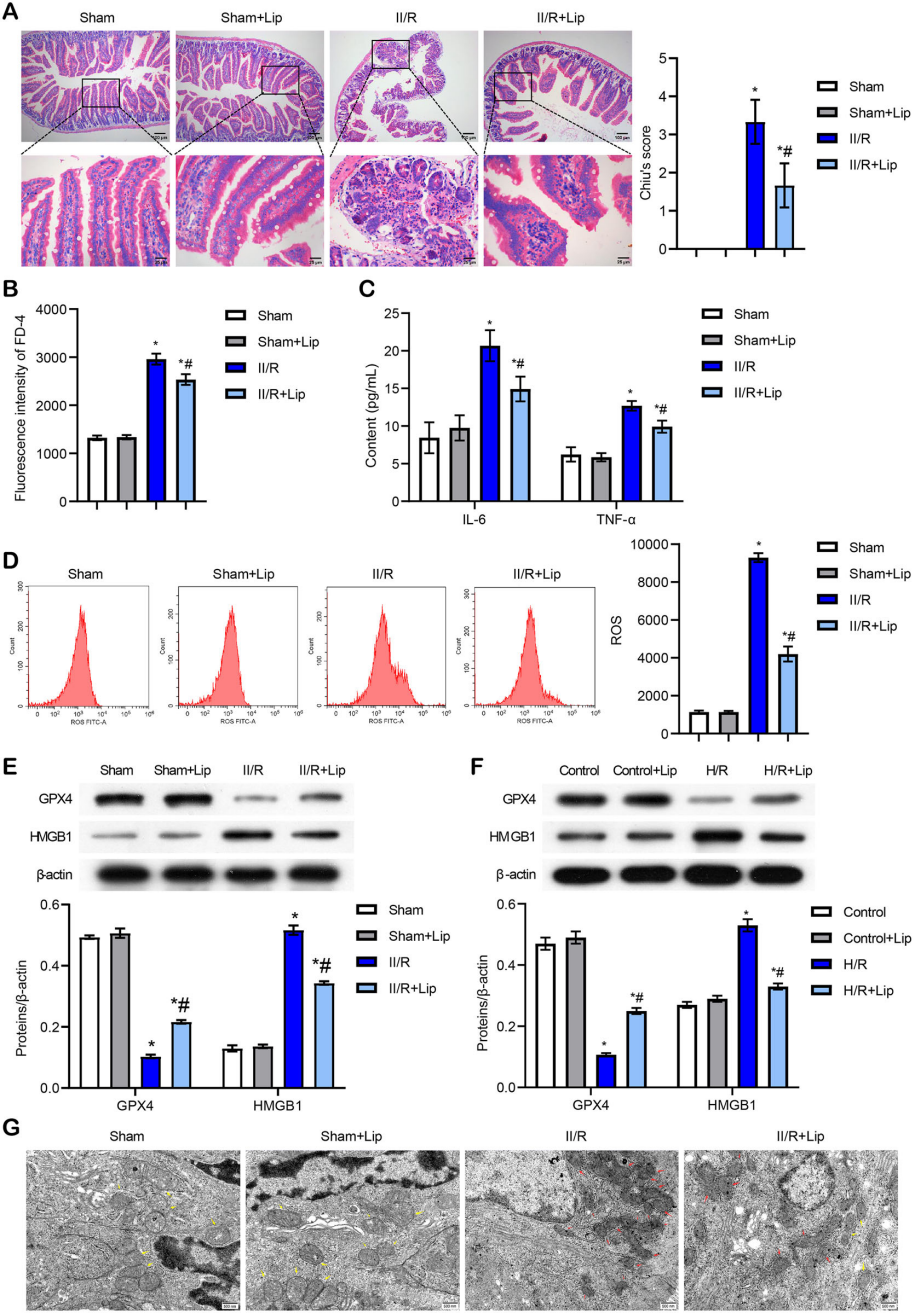

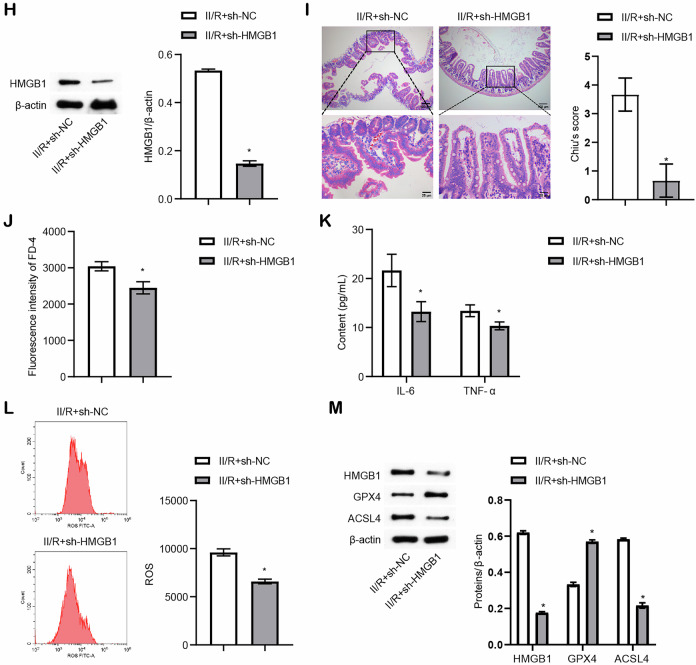
Lip or HMGB1 inhibited ferroptosis induced by II/R. For animal experiments, 10 mg/kg Lip was injected intraperitoneally 4 h before and after ischemia to evaluate its effect on II/R-induced ferroptosis [4, 52]. For cell experiments, Caco-2 cells were treated with Lip at a final concentration of 200 nM for 12 h before inducing hypoxia. **A** HE staining illustrating intestinal tissue damage and Chiu’s histological score for intestinal injury. Scale bar = 100 μm (100×), 25 μm (400×). **B** Intestinal permeability was determined using fluorescein isothiocyanate-dextran (FD-4). **C** ELISA analysis of TNF-α and IL-6 levels. **D** ROS levels were assessed by flow cytometry. **E**, **F** Western blot analysis of GPX4 and HMGB1 expression. **G** TEM analysis of ferroptosis. Red arrows indicate ferroptotic mitochondria, and yellow arrows indicate normal mitochondria. Scale bar = 500 nm. **H** Western blot detection of HMGB1 expression in intestinal tissue. **I** HE staining and Chiu’s histological score for intestinal injury. Scale bar = 100 μm (100×), 25 μm (400×). **J** Intestinal permeability assessment. **K** ELISA detection of inflammatory cytokines TNF-α and IL-6. **L** Lipid ROS levels in tissues were analyzed by flow cytometry. **M** Western blot analysis of GPX4, HMGB1, and ACSL4 expression in tissues. *P < 0.05 vs Sham or Control, ^#^*P* < 0.05 vs II/R or H/R. For animal experiments, *n* = 6 mice per group. For Western blot experiments, *n* = 3.

First, we used a bioinformatic database to screen for genes that were abnormally expressed in II/R. The top 20 differentially upregulated and down-regulated genes were screened in the GSE37013 database between the Control and II/R groups. Difference analysis was conducted using the limma package in R. The screening criteria are as follows: The cutoff was |logFC|>log_2_(1.5) and *P* < 0.05. The top 20 differentially upregulated and downregulated genes were presented in Fig. S1A. Ferroptosis, an essential form of cell death, has been implicated in exacerbating II/R injuries [19]. Since previous studies have associated HMGB1 and SREBF2 with ferroptosis [20, 21], we wanted to further verify whether there could be an association between II/R with ferroptosis and the HMGB1/SREBF2 axis. As shown in Fig. S1B, HMGB1 and SREBF2 were significantly upregulated in II/R tissues. To explore the molecular mechanisms underlying II/R-induced ferroptosis, we constructed an II/R model and treated animals with the ferroptosis inhibitor Lip, and the results showed that the expression of SREBF2 and HMGB1 increased significantly in the II/R-2h, II/R-4h, and II/R-8h groups compared to the Sham group, with the highest levels observed at 4 h of reperfusion (Fig. S1C). Thus, subsequent experiments were conducted using 4 h of reperfusion. The flowchart of animal handling is shown in Fig. S1D. Lip, a potent and specific ferroptosis inhibitor, significantly attenuated II/R-induced intestinal villus damage, reduced Chiu’s histological score for intestinal injury, and improved intestinal permeability, as demonstrated by HE staining and decreased serum FD-4 levels (Fig. 1A, B). TNF-α and IL-6 levels were significantly elevated in the II/R group, but Lip treatment significantly reduced IL-6 levels (Fig. 1C). Flow cytometry analysis further revealed that Lip effectively mitigated II/R-induced ROS accumulation (Fig. 1D). Additionally, Lip was shown to rescue the effects of II/R by suppressing GPX4 expression, a key enzyme involved in ferroptosis inhibition [10]. Importantly, II/R significantly induced HMGB1 expression, which was suppressed by ferroptosis inhibition through Lip treatment (Fig. 1E). These in vivo findings were corroborated by H/R experiments, which demonstrated that H/R significantly induced HMGB1 expression and suppressed GPX4 expression, both of which were reversed by Lip treatment (Fig. 1F). Characteristic ferroptosis-related mitochondrial changes, including rupture of the outer mitochondrial membrane and reduction in mitochondrial cristae, were observed in the II/R group. However, these structural abnormalities could be alleviated by Lip treatment (Fig. 1G). Western blot analysis showed that HMGB1 expression in intestinal tissue was significantly reduced in the II/R+sh-HMGB1 group compared with the II/R+sh-NC group (Fig. 1H). In addition, HE staining revealed severe intestinal tissue damage and pronounced inflammatory infiltration in the II/R+sh-NC group, accompanied by a high Chiu’s histological score for intestinal injury. HMGB1 interference alleviated this damage and reduced Chiu’s score (Fig. 1I). Furthermore, intestinal permeability, TNF-α and IL-6 levels, and lipid ROS levels were significantly decreased in the II/R+sh-HMGB1 group compared with the II/R+sh-NC group (Fig. 1J–L). Notably, GPX4 expression was increased in the II/R+sh-HMGB1 group, while HMGB1 and ACSL4 expression levels were decreased compared with the II/R+sh-NC group (Fig. 1M). Collectively, these results demonstrate that HMGB1 is highly expressed in ferroptosis-related pathways during II/R injury, and its inhibition, or suppression of ferroptosis, effectively mitigates II/R-induced damage.

### Knockdown of HMGB1 inhibited ferroptosis induced by H/R in Caco-2 cells

**Fig. 2:**
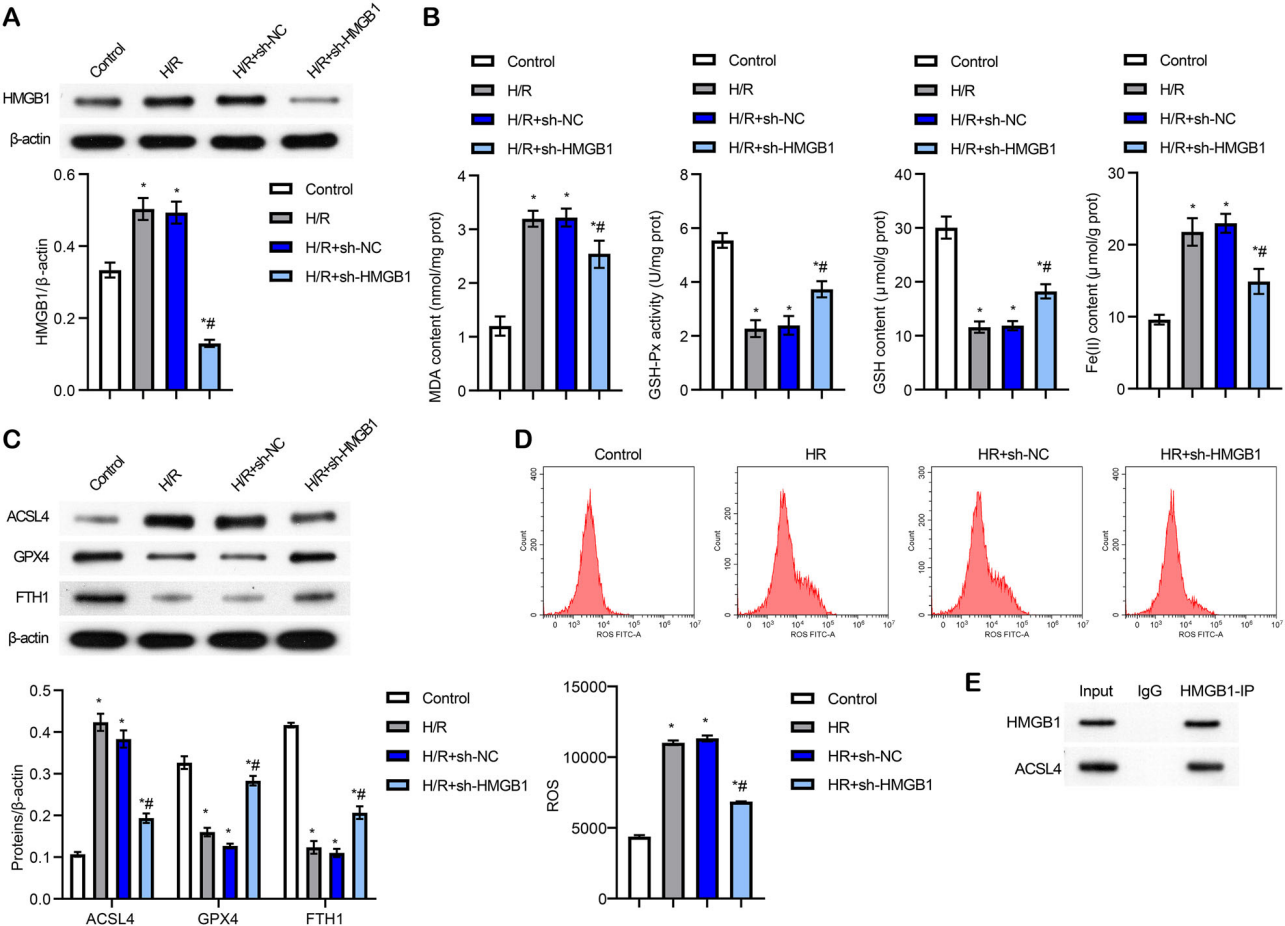
Knockdown of HMGB1 inhibited ferroptosis induced by H/R in Caco-2 cells. **A** Western blot analysis of HMGB1 expression. **B** Biochemical assays measuring MDA content, GSH-Px activity, GSH content, and Fe (II) levels. **C** Western blot detection of ferroptosis-related protein levels (ACSL4, GPX4, and FTH1). **D** ROS levels assessed by flow cytometry. **E** Co-IP analysis demonstrating the interaction between HMGB1 and ACSL4. For cell experiments, *n* = 3. For western blot experiments, *n* = 3. *P < 0.05 vs H/R, ^#^*P* < 0.05 vs H/R+sh-NC.

To investigate the role of HMGB1 in H/R-induced ferroptosis in Caco-2 cells, we transfected cells with sh-HMGB1 or its control, sh-NC. The efficiency of HMGB1 knockdown was validated in vitro, as shown in Fig. S2A, confirming the successful silencing of HMGB1. Using this model, we observed that sh-HMGB1 significantly reduced the H/R-induced expression of HMGB1 (Fig. 2A). Knockdown of HMGB1 increased GSH-Px activity and GSH levels while reducing MDA and Fe (II) levels in H/R-treated Caco-2 cells (Fig. 2B). Additionally, the expression of ferroptosis-associated proteins, including ACSL4, GPX4, and FTH1, was reversed following HMGB1 knockdown (Fig. 2C). Flow cytometry analysis demonstrated that HMGB1 silencing significantly decreased H/R-induced ROS levels in Caco-2 cells (Fig. 2D). Previous studies have shown that cytosolic HMGB1 induces ferroptosis by interacting with ACSL4, a key driver of ferroptosis, during renal ischemia/reperfusion [22]. We then used co-IP and identified the interaction between HMGB1 and ACSL4 (Fig. 2E). Taken together, these results indicate that HMGB1 knockdown inhibits ferroptosis induced by H/R in Caco-2 cells.

### SREBF2 directly regulated HMGB1 expression to promote H/R-induced ferroptosis in Caco-2 cells

**Fig. 3:**
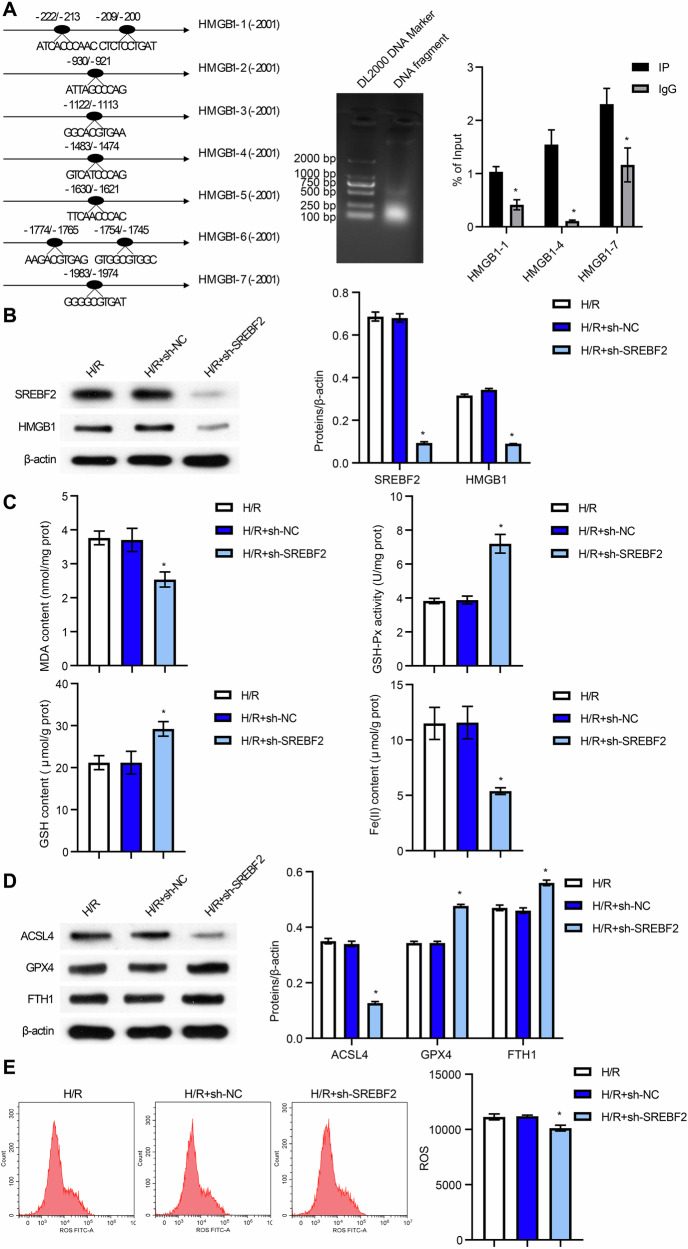
SREBF2 directly regulated HMGB1 expression to promote H/R-induced ferroptosis in Caco-2 cells. **A** Ch-IP analysis verified the binding of SREBF2 to the HMGB1 promoter (*P < 0.05 vs IP). **B** Western blot detection of SREBF2 and HMGB1 expression levels. **C** Biochemical assays measuring MDA content, GSH-Px activity, GSH content, and Fe (II) levels. **D** Western blot analysis of ferroptosis-related proteins (ACSL4, GPX4, and FTH1). **E** Flow cytometry analysis of ROS levels. For cell experiments, *n* = 3. For Western blot experiments, *n* = 3. *P < 0.05 vs H/R+sh-NC.

Next, we investigated the regulatory mechanisms underlying HMGB1-mediated H/R-induced ferroptosis in Caco-2 cells, and Pearson’s correlation analysis revealed a positive correlation between HMGB1 and SREBF2 expression (Fig. S2B). Moreover, SREBF2 levels were significantly elevated in H/R-treated Caco-2 cells (Fig. S2C). The binding sites were predicted using JASPAR (https://jaspar.genereg.net/analysis), which suggested direct interactions between SREBF2 and the HMGB1 promoter region. ChIP confirmed that SREBF2 directly binds to the HMGB1 promoter, thereby regulating its expression (Fig. S2D and Fig. 3A). SREBF2 was knocked down in Caco-2 cells to validate this regulatory relationship, and the knockdown efficiency was confirmed (Fig. S2E). In H/R-treated Caco-2 cells, sh-SREBF2 significantly reduced the expression of SREBF2 and HMGB1 (Fig. 3B), and this knockdown also increased GSH-Px activity and GSH levels while reducing MDA and Fe (II) levels (Fig. 3C). Consistently, sh-SREBF2 reversed the expression of ferroptosis-related proteins, including ACSL4, GPX4, and FTH1 (Fig. 3D). Flow cytometry further demonstrated that SREBF2 knockdown decreased ROS levels in H/R-treated Caco-2 cells (Fig. 3E). These findings establish that SREBF2 directly regulates HMGB1 expression, promoting H/R-induced ferroptosis in Caco-2 cells.

### BMSC-EVs suppressed the SREBF2/HMGB1 axis and alleviated H/R-induced ferroptosis in Caco-2 cells

**Fig. 4:**
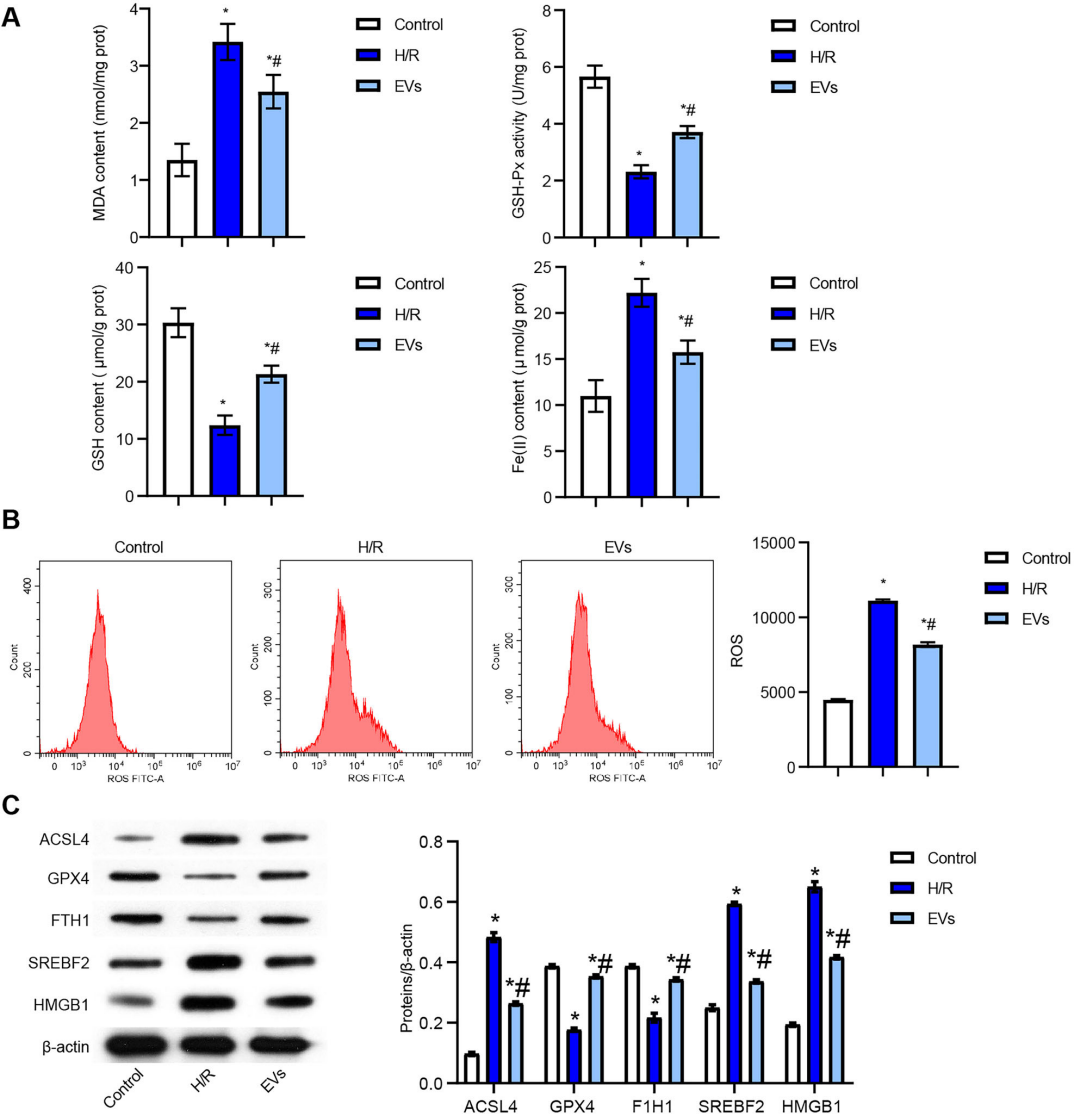
BMSC-EVs suppressed SREBF2/HMGB1 axis and alleviated H/R-induced ferroptosis in Caco-2 cells. **A** Biochemical assays measuring MDA content, GSH-Px activity, GSH content, and Fe (II) levels. **B** ROS levels analyzed by flow cytometry. **C** Western blot detection of ferroptosis-related proteins (ACSL4, GPX4, FTH1, SREBF2, and HMGB1). For cell experiments, *n* = 3. For Western blot experiments, *n* = 3. *P < 0.05 vs Control, ^#^*P* < 0.05 vs H/R.

To confirm the differentiation potential of BMSCs, we assessed their tri-lineage differentiation capabilities. The osteogenic, lipogenic and chondrogenic differentiation of BMSCs were verified (Fig. S3A–C), which confirmed that BMSCs possess the ability for tri-lineage differentiation. Given their ideal size and inherent biocompatibility, EVs are emerging as a promising therapeutic vector [23]. Then, we examined the effects of BMSC-EVs on H/R-mediated ferroptosis in Caco-2 cells. BMSC-EVs were successfully isolated from BMSCs. TEM revealed that the purified BMSC-EVs exhibited a characteristic saucer-like morphology with diameters ranging from 100 to 200 nm (Fig. S3D, E). Western blot showed that BMSC-EVs were expressed and enriched for markers CD63, TSG101, CD81, and CD9, but not expressed for marker Calnexin (Fig. S3F). These observations indicated that the vesicles were BMSC-EVsA subsequent phagocytosis experiment demonstrated that Caco-2 cells effectively internalized BMSC-EVs (Fig. S3G). Treatment with BMSC-EVs significantly increased GSH-Px activity and GSH levels while reducing MDA, Fe (II), and ROS levels in H/R-induced Caco-2 cells (Fig. 4A, B). In line with these findings, BMSC-EVs reversed the expression of ferroptosis-related proteins ACSL4, GPX4, and FTH1 (Fig. 4C), suggesting that BMSC-EVs alleviated H/R-induced ferroptosis. Notably, BMSC-EVs also suppressed the expression of SREBF2 and HMGB1 (Fig. 4C). Overall, these results demonstrate that BMSC-EVs alleviate H/R-induced ferroptosis in Caco-2 cells by suppressing the SREBF2/HMGB1 axis.

### miR-378a-3p regulated the SREBF2/HMGB1 axis

**Fig. 5:**
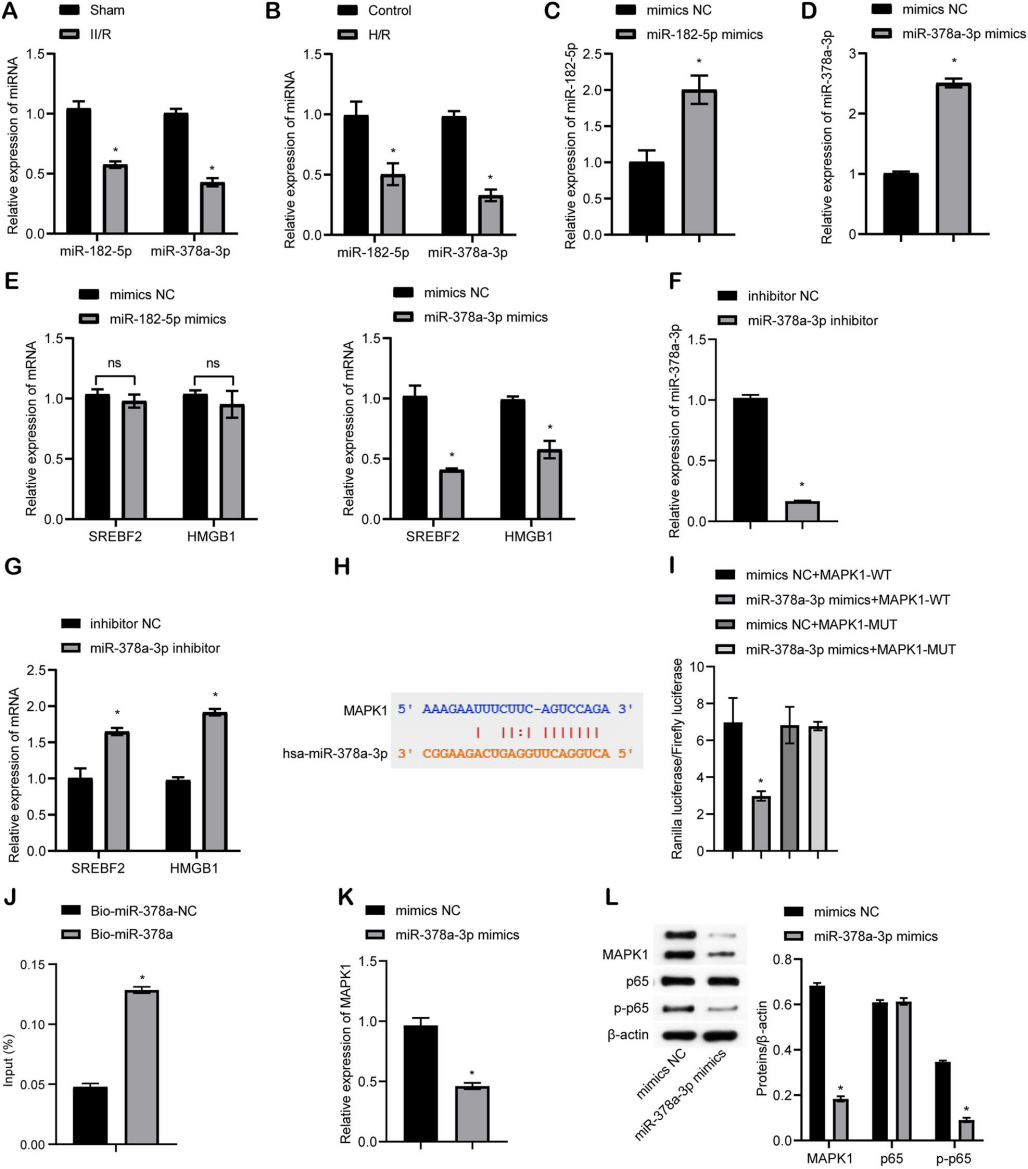
miR-378a-3p regulated the SREBF2/HMGB1 axis. **A**–**D** qRT-PCR analysis of miR-182-5p and miR-378a-3p expression. **E** qRT-PCR assessment of SREBF2 and HMGB1 expression levels. **F** and **G** qRT-PCR analysis of miR-378a-3p, SREBF2 and HMGB1 expression levels. **H** Bioinformatics prediction of miR-378a-3p binding sites on MAPK1. **I** Dual-luciferase reporter assay confirming the interaction between hsa-miR-378a-3p and MAPK1. **J** RNA pull-down assay validating the interaction between hsa-miR-378a-3p and MAPK1. **K** qRT-PCR detection of MAPK1 expression. **L** Western blot analysis of MAPK1, p65, and p-p65 expression. For animal experiments, *n* = 6 mice per group. For cell experiments, *n* = 3. For western blot experiments, *n* = 3. *P < 0.05 vs Sham, Control, mimics NC, inhibitor NC or Bio-miR-378a-NC.

To further investigate the mechanism by which BMSC-EVs mitigate II/R-induced ferroptosis, we first screened for downregulated miRNAs in II/R, among which miR-182 and miR-378 were found to be notably decreased in the model group. Previous studies have shown that miR-182 upregulation reduces intestinal injury, autophagy, and Deptor expression while enhancing mTOR activity following II/R [24, 25]. Similarly, miR-378 downregulation has been associated with increased intestinal injury, while its overexpression inhibits caspase-3 activation and contributes to a protective effect in II/R [26]. Based on the prediction that miR-182-5p and miR-378a-3p may target SREBF2, we hypothesized that these miRNAs could regulate the SREBF2/HMGB1 axis. To explore this, we examined the expression of miR-182-5p and miR-378a-3p in II/R cells. Figure 5A and B show the differentially expressed miRNAs in the Sham versus II/R groups and the Control versus H/R groups, respectively, showing that miR-182-5p and miR-378a-3p expression was significantly reduced in both II/R and H/R groups, and the overexpression of miR-182-5p and miR-378a-3p was successfully validated (Fig. 5C, D). Further analysis revealed that overexpression of miR-378a-3p significantly decreased the expression of SREBF2 and HMGB1. In contrast, miR-182-5p overexpression did not significantly change SREBF2 or HMGB1 expression (Fig. 5E). Based on the above results, we selected miR-378a-3p for subsequent studies. miR-378a-3p knockdown was successfully validated (Fig. 5F), and could significantly increase the expression of SREBF2 and HMGB1 (Fig. 5G). Bioinformatics prediction identified potential binding sites for miR-378a-3p on SREBF2 (Fig. S4A). However, dual-luciferase reporter assays indicated that miR-378a-3p does not directly target SREBF2 (Fig. S4B). Previous studies have shown that miR-378a-3p targets MAPK1 to regulate cardiac cell hypertrophy [27]. Moreover, inhibition of the p38 MAPK/NF-κB signaling pathway protects the intestinal barrier from ischemia/reperfusion-induced injury [28]. Additionally, NF-κB has been demonstrated to directly bind to the SREBF2 promoter, thereby activating SREBF2 expression [29]. These findings led us to hypothesize that miR-378a-3p might suppress the SREBF2/HMGB1 axis by targeting the MAPK1/NF-κB pathway. Bioinformatics prediction confirmed binding sites between miR-378a-3p and MAPK1 (Fig. 5H). This interaction was further validated by dual-luciferase reporter assays (Fig. 5I) and RNA pull-down experiments (Fig. 5J). Overexpression of miR-378a-3p significantly reduced MAPK1 mRNA and protein expression, as well as the protein levels of p-p65, a key component of the NF-κB signaling pathway (Fig. 5K, L). Collectively, these results demonstrate that miR-378a-3p regulates the SREBF2/HMGB1 axis by targeting the MAPK1/NF-κB pathway.

### miR-378a-3p derived form BMSC-EVs regulated SREBF2/HMGB1 axis to inhibit H/R-induced ferroptosis in Caco-2 cells

**Fig. 6:**
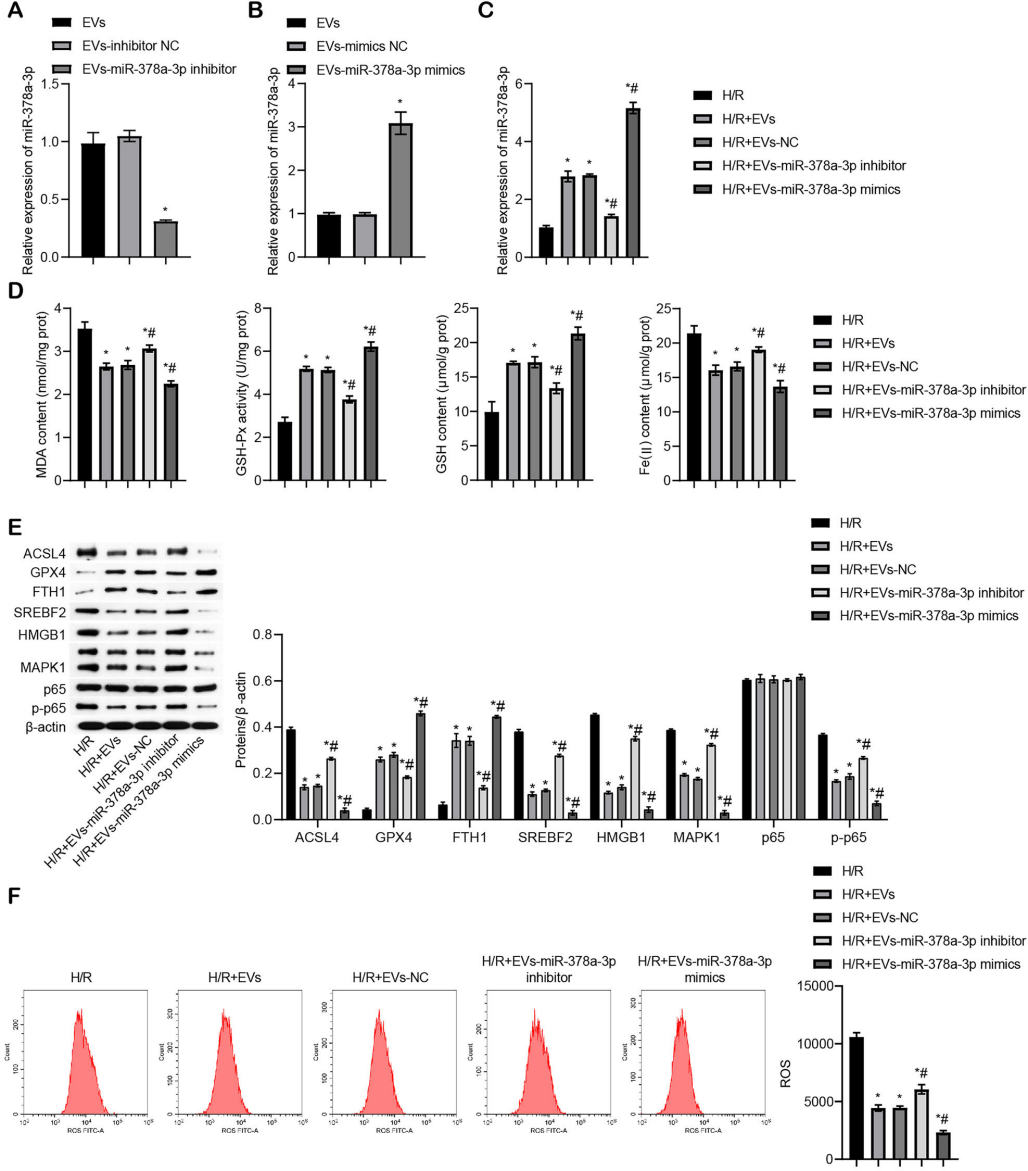
miR-378a-3p derived from BMSC-EVs regulated SREBF2/HMGB1 axis to inhibit H/R-induced ferroptosis in Caco-2 cells. **A** miR-378a-3p expression determined by qRT-PCR (*P < 0.05 vs EVs-inhibitor NC). **B** qRT-PCR analysis of miR-378a-3p expression (*P < 0.05 vs EVs-mimics NC). **C** qRT-PCR detection of miR-378a-3p expression (*P < 0.05 vs H/R, ^#^*P* < 0.05 vs H/R+EVs-NC). **D** Biochemical analysis of MDA content, GSH-Px activity, GSH content, and Fe (II) levels. **E** Western blot detection of ferroptosis-related proteins ACSL4, GPX4, FTH1, SREBF2, and HMGB1, along with MAPK1, p65, and p-p65 levels. **F** ROS levels measured by flow cytometry. For cell experiments, *n* = 3. For Western blot experiments, *n* = 3. *P < 0.05 vs H/R, ^#^*P* < 0.05 vs H/R+EVs-inhibitor NC.

Previous research has demonstrated that BMSC-derived EVs can influence rheumatoid arthritis by delivering miR-378a-5p, where miR-378a-5p was highly expressed in BMSC-derived EVs but showed low expression in synovial tissue of rheumatoid arthritis patients [30]. Another study revealed that miR-378 regulates the chondrogenesis of BMSCs [31], suggesting an essential role for miR-378 in the functional regulation of BMSCs. To further investigate the role of miR-378a-3p, we measured its expression in BMSC-EVs. The expression of miR-378a-3p was significantly higher in the BMSC-EVs group compared to the BMSC group (Fig. S5). To modulate miR-378a-3p expression, we employed specific inhibitors and mimics. miR-378a-3p expression was reduced in the EVs-miR-378a-3p inhibitor group compared to the EVs-inhibitor NC group (Fig. 6A). Conversely, miR-378a-3p expression was elevated in the EVs-miR-378a-3p mimics group compared to the EVs-mimics NC group, confirming the successful transfection of the mimics (Fig. 6B). Additionally, in the H/R+EVs group, miR-378a-3p expression was higher than in the H/R group. In the EV-treated groups, miR-378a-3p expression decreased in the H/R+EVs-miR-378a-3p inhibitor group and increased in the H/R+EVs-miR-378a-3p mimics group compared to the H/R+EVs-NC group (Fig. 6C). Furthermore, functional analyses showed that compared to the H/R group, MDA and Fe(II) levels were reduced, while GSH and GSH-Px levels were elevated in the H/R+EVs group. miR-378a-3p inhibition reversed these changes, whereas its overexpression enhanced the effects induced by EVs (Fig. 6D). Similarly, in the H/R+EVs group, ACSL4, HMGB1, SREBF2, MAPK1, and p-p65 levels were reduced, while FTH1 and GPX4 levels were increased compared to the H/R group. Inhibition of miR-378a-3p reversed these effects, while overexpression further enhanced the regulatory impact of EVs (Fig. 6E). ROS levels were also significantly decreased in the H/R+EVs group compared to the H/R group. miR-378a-3p inhibition increased lipid ROS levels, whereas overexpression of miR-378a-3p suppressed ROS levels (Fig. 6F). Collectively, these findings demonstrate that miR-378a-3p derived from BMSC-EVs regulates the SREBF2/HMGB1 axis and inhibits H/R-induced ferroptosis in Caco-2 cells.

### miR-378a-3p derived from BMSC-EVs alleviated II/R-induced ferroptosis via SREBF2/HMGB1 axis

**Fig. 7:**
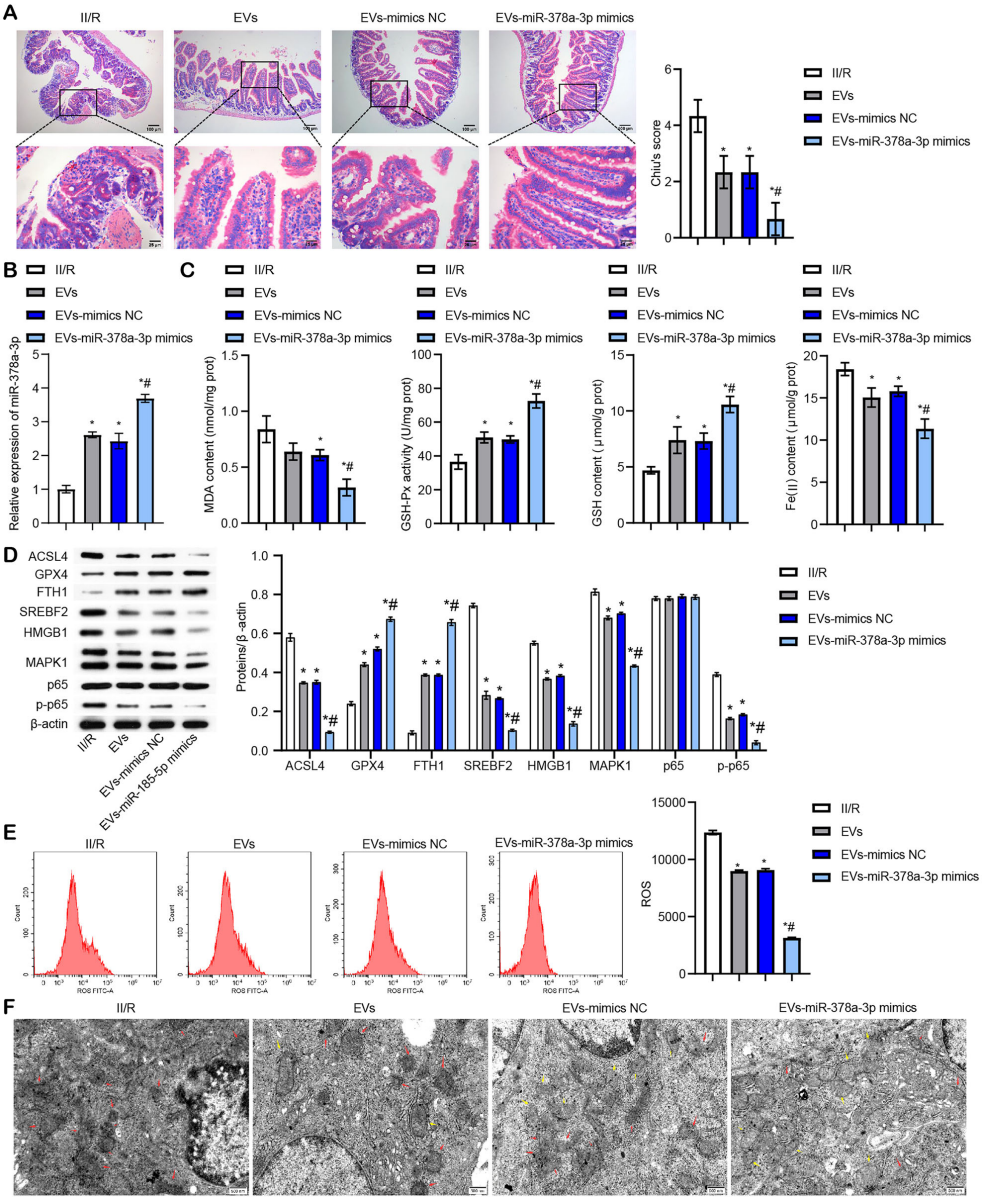
miR-378a-3p derived from BMSC-EVs alleviated II/R-induced ferroptosis via SREBF2/HMGB1 axis. **A** HE staining of intestinal tissue damage and Chiu’s histological score for intestinal injury. Scale bar = 100 μm (100×), 25 μm (400×). **B** miR-378a-3p expression monitored through qRT-PCR. **C** Biochemical analysis of MDA content, GSH-Px activity, GSH content, and Fe (II) levels. **D** Western blot detection of ferroptosis-related proteins ACSL4, GPX4, FTH1, SREBF2, HMGB1, MAPK1, p65 and p-p65 levels. **E** ROS levels measured by flow cytometry. **F** TEM analysis of ferroptosis. Red arrows indicate ferroptotic mitochondria, and yellow arrows indicate normal mitochondria. Scale bar = 500 nm. For animal experiments, *n* = 3. For Western blot experiments, *n* = 3. *P < 0.05 vs II/R, ^#^*P* < 0.05 vs EVs-mimics NC.

To further evaluate the potential role of miR-378a-3p in regulating II/R-induced ferroptosis in vivo, we treated II/R mice with BMSC-EVs overexpressing miR-378a-3p. Treatment with EVs-miR-378a-3p mimics alleviated II/R-induced intestinal mucosal injury and reduced Chiu’s score, as demonstrated by HE staining (Fig. 7A), indicating that miR-378a-3p derived from BMSC-EVs mitigated II/R injury. Figure 7B shows that EVs-miR-378a-3p mimics significantly elevated miR-378a-3p expression. Additionally, EVs-miR-378a-3p mimics notably increased GSH-Px activity and GSH levels while reducing MDA and Fe (II) levels in the intestinal tissues of II/R mice (Fig. 7C). Consistent with these findings, treatment with EVs-miR-378a-3p mimics restored the expression of ACSL4, GPX4, and FTH1 (Fig. 7D), and decreased ROS levels in the intestinal tissues of II/R mice (Fig. 7E), indicating that the mimics alleviated II/R-induced ferroptosis in vivo. Furthermore, EVs-miR-378a-3p mimics significantly suppressed the expression of SREBF2, HMGB1, MAPK1, and p-p65 in the intestinal tissues of II/R mice (Fig. 7D). TEM revealed that EVs-miR-378a-3p mimics attenuated II/R-induced ferroptosis-related morphological changes, including rupture of the outer mitochondrial membrane and reduction in mitochondrial cristae(Fig. 7F). These results suggest that miR-378a-3p derived from BMSC-EVs alleviates II/R-induced ferroptosis by modulating the SREBF2/HMGB1 axis.

## Discussion

II/R injury is a complex pathophysiological process associated with high mortality and significant challenges in clinical management [32, 33]. In this study, we identified a novel miR-378a-3p/SREBF2/HMGB1 axis that plays a key role in the mitigation of II/R-induced ferroptosis by BMSC-EVs. Mechanistically, miR-378a-3p derived from BMSC-EVs regulates the SREBF2/HMGB1 axis to alleviate ferroptosis induced by II/R. These findings broaden the research on the pathological mechanism of II/R injury and provide a theoretical basis for developing potential targets for treating ferroptosis caused by II/R.

The process of II/R is dynamic and involves multiple complex mechanisms of cell death. II/R injury has been linked to apoptosis, necroptosis, and autophagy [34–36]. Recently, ferroptosis has also been implicated in II/R injury [4]. During reperfusion, ferroptosis may act as a lethal process, exacerbating II/R injury. Emerging studies have shown that capsiate, a metabolite of the gut microbiota, upregulates GPX4 expression by activating transient receptor potential cation channel subfamily V member 1 (TRPV1), thereby inhibiting ferroptosis and protecting the intestine from ischemia/reperfusion injury [19]. In line with these findings, our study also demonstrated that inhibiting ferroptosis alleviates intestinal mucosal injury during II/R. These observations suggest that targeting ferroptosis inhibition may represent a promising therapeutic approach for managing II/R injury.

HMGB1 plays a central role in initiating and amplifying immune responses, which then promotes cell proliferation and contributes to the pathological processes of ischemia/reperfusion injury in organs such as the kidney, heart, brain, and liver [37]. Furthermore, HMGB1 is an important regulator of ferroptosis in various diseases as it can modulate ferroptosis through pathways such as nuclear factor erythroid-related factor 2 (Nrf2) or RAS-JNK/p38 [22]. Although the mechanisms underlying HMGB1’s biological functions are still being elucidated, oxidative stress is a pivotal regulator of HMGB1 translocation, release, and activity. These processes are linked to various forms of cell death, including necrosis, apoptosis, autophagic cell death, and pyroptosis [38]. HMGB1 regulates oxidative stress by inhibiting the Nrf2 pathway, whereas suppression of HMGB1 expression and cytoplasmic translocation activates Nrf2 and its downstream antioxidant genes [39]. Recent research has demonstrated that cytoplasmic HMGB1 can exacerbate inflammation-associated cellular injury by activating renal tubular ferroptosis following I/R injury, highlighting HMGB1 as a promising therapeutic target for acute kidney injury (AKI) [22]. Given its regulatory role in ferroptosis, HMGB1 may also hold therapeutic potential for other human diseases associated with ferroptosis [40]. In this present study, we found that inhibiting HMGB1 expression reduced ferroptosis levels in intestinal epithelial cells during H/R. Our findings suggest that HMGB1 participates in H/R-induced ferroptosis by modulating GPX4 activity. However, further research is required to clarify the complex interactions between these cell death processes.

Since intestinal ischemia is clinically challenging to prevent, much of the research in this field focuses on developing novel treatments for post-ischemic injury during the reperfusion period, such as antioxidants, free radical scavengers, and hydrogen-rich saline. Mesenchymal stem cells (MSCs) are the most extensively studied stem cells and have been recognized as a promising therapeutic approach for various diseases [41, 42], including intestinal ischemic conditions. However, the unclear therapeutic mechanisms of MSC-based treatments have limited their widespread application. Recent studies have shown that exosomes derived from human umbilical cord blood mesenchymal stem cells improve intestinal epithelial barrier function during II/R injury by modulating the Snail/Claudins signaling pathway through miR-34a/c-5p and miR-29b-3p [43]. In the current study, we demonstrated that BMSC-EVs alleviate injury in intestinal epithelial cells following hypoxia and reoxygenation, significantly reducing HMGB1 expression and ferroptosis levels. However, the precise mechanisms underlying these effects warrant further investigation.

SREBF2 is an important player in adipogenesis and serves as a master regulator of cholesterol synthesis. As a transcription factor, SREBF2 is also implicated in various physiological and pathological processes, including ROS generation, endoplasmic reticulum stress, cell apoptosis, and autophagy [44]. Additionally, SREBF2 can induce transferrin and inhibit ferroptosis in circulating melanoma cells [21]. However, its role in II/R injuries has not been previously reported. In this study, we demonstrated for the first time that miR-378a-3p derived from BMSC-EVs effectively alleviates II/R-induced ferroptosis via the SREBF2/HMGB1 axis. BMSC-EVs are extensively studied and known to carry diverse bioactive molecules, such as proteins and miRNAs, that influence recipient cell behavior through intercellular communication [45]. With its demonstrated safety and efficacy in clinical applications, BMSC-EVs represent a promising candidate for therapeutic strategies [46]. EVs serve as essential mediators of cell-to-cell communication by transporting bioactive substances. We focused on miR-378a-3p due to its documented role in biological processes such as cell apoptosis, proliferation, and differentiation [47]. Our findings confirm that miR-378a-3p derived from BMSC-EVs is a key miRNA that alleviates II/R-induced ferroptosis. Previous studies have highlighted the involvement of miR-182-5p and miR-378a-3p in regulating ferroptosis in I/R-induced renal injury [48]. Additionally, miR-378a-3p/SLC7A11 has been shown to regulate ferroptosis in nerve injury caused by lead exposure, with miR-378a-3p inhibition reversing lead-induced decreases in GSH levels and increases in lipid ROS levels [49]. In the present study, we demonstrated that miR-378a-3p derived from BMSC-EVs regulates the SREBF2/HMGB1 axis to inhibit H/R-induced ferroptosis in Caco-2 cells. Furthermore, miR-378a-3p derived from BMSC-EVs alleviated II/R-induced ferroptosis via the SREBF2/HMGB1 axis. SREBF2 acts as a transcription factor for the ferroptosis regulator HMGB1, which negatively influences GPX4 and is instrumental in II/R-induced ferroptosis [50]. Therefore, miR-378a-3p may inhibit the HMGB1/GPX4 axis activation by regulating SREBF2, offering a potential therapeutic strategy for II/R injuries.

In conclusion, our study demonstrates that BMSC-EVs regulate the SREBF2/HMGB1 axis through the delivery of miR-378a-3p, effectively inhibiting ferroptosis during II/R injury. These findings provide valuable insights into the molecular mechanisms underlying the occurrence and progression of II/R injury and propose a novel therapeutic approach for its treatment.

## Materials and methods

### Animal model of II/R

Eight-week-old male C57BL/6 mice were purchased from Hunan SJA Laboratory Animal Co., Ltd, and the II/R model (II/R group) was established through midline laparotomy. To determine the optimal reperfusion time, the mice were randomly assigned to five groups: Sham, II/R-1h (45 min of ischemia followed by 1 h of reperfusion), II/R-2h (45 min of ischemia followed by 2 h of reperfusion), II/R-4h (45 min of ischemia followed by 4 h of reperfusion), and II/R-8h (45 min of ischemia followed by 8 h of reperfusion), with six mice per group. Briefly, their superior mesenteric artery (SMA) was identified and occluded using a microvascular clamp 45 min after laparotomy, and reperfusion was allowed for 4 h by removing the clamp [51].

The mice were anesthetized intraperitoneally with pentobarbital (50 mg/kg body weight). In the Sham group, the SMA was separated without occlusion, and the surgical procedure was otherwise identical to that of the II/R groups. To investigate the role of ferroptosis in II/R injury, 10 mg/kg liproxstatin-1 (Lip, S7699, Selleck, USA) was administered intraperitoneally 4 h before and after ischemia in the Sham+Lip and II/R+Lip groups [4, 52]. For BMSC-EV treatment, II/R mice received caudal vein injections of 30 μg BMSC-EVs daily for three consecutive days post-reperfusion. In parallel, the II/R group received equivalent volumes of PBS without BMSC-EVs [53]. In contrast, to evaluate the effect of HMGB1, sh-HMGB1 or sh-NC lentivirus (1 × 10^9^ viral particles per mouse) were injected in the tail vein of mice in the II/R group with seven days before II/R modeling [54]. These groups were designated as II/R+sh-HMGB1 and II/R+sh-NC, respectively. Additionally, to assess the role of miR-378a-3p, mice were divided into the following groups: II/R, EVs, EVs-mimics NC, and EVs-miR-378a-3p mimics. Mice were sacrificed with pentobarbital three days after II/R modeling, and small intestines were collected for analysis.

### Cell hypoxia/reoxygenation (H/R) model and cell culture

Caco-2 cells (CL-0050, Procell, China) were cultured in DMEM supplemented with non-essential amino acids, fetal bovine serum, and penicillin/streptomycin at 37 °C in a 5% CO_2_ atmosphere. Bone marrow mesenchymal stem cells (BMSC, AW-YCH025), human adipose mesenchymal stem cells (AMSC, AW-YCH124), and human umbilical cord mesenchymal stem cells (UCMSC, AW-YCH122) were purchased from Abiowell and maintained in either mesenchymal stem cell medium (AW-MC025, Abiowell) or serum-free medium for primary mesenchymal stem cells (AW-MC036, Abiowell). All cells were recently authenticated via short tandem repeat (STR) profiling and confirmed to be free from mycoplasma contamination.

To establish the H/R model, Caco-2 cells were cultured in a microaerophilic system (Thermo Fisher Scientific, USA) equilibrated to 5% CO_2_, 1% O_2_, and 94% N_2_ for 6 h to induce hypoxia, followed by 24 h under normoxic conditions for reoxygenation [4, 52]. Before hypoxia induction, Caco-2 cells were treated with Lip at a final concentration of 200 nM for 12 h. For EV treatments, BMSC-EVs were added to fetal bovine serum depleted of EVs at a concentration of 100 µg/mL for 24 h following hypoxia.

Gene knockdown and overexpression studies were performed to investigate the roles of HMGB1, SREBF2, miR-378a-3p, and miR-182-5p. The knockdown was achieved using shRNAs (sh-HMGB1, sh-SREBF2) and miRNA inhibitors (miR-378a-3pinhibitor), while overexpression was induced using overexpression vectors (oe-SREBF2) and miRNA mimics (miR-182-5p mimics, miR-378a-3p mimics). All constructs and reagents were synthesized by HonorGene (Changsha, China). Caco-2 cells and BMSCs were cultured to 60–80% confluency before transfection. Transfection complexes were prepared using Lipofectamine 3000 (Invitrogen, USA) according to the manufacturer’s instructions. Briefly, shRNAs, miRNA inhibitors, overexpression vectors, or miRNA mimics were mixed with Lipofectamine 3000 in serum-free medium and incubated at room temperature for 20 min to form transfection complexes. These complexes were added to the cell cultures and gently agitated to ensure uniform distribution. The transfected cells were incubated at 37 °C in a 5% CO_2_ incubator for 6 h after which the medium was replaced with a fresh complete culture medium.

### Alizarin red staining

BMSCs were cultured in an osteogenic induction medium, and the medium was replaced every 2–3 days. After 21 days of continuous induction, the osteogenic differentiation status of BMSCs was examined under a microscope. The cells in six-well plates were washed twice with PBS, fixed with 4% paraformaldehyde for 30 min, and washed again with PBS. Alizarin Red S staining solution (ALIR-10001, CYAGEN) was then applied, and the cells were stained at room temperature for 30 min. Following three washes with PBS, the osteogenic staining was visualized under a microscope.

### Oil-red O staining

BMSCs were induced with an adipogenic induction medium, beginning with Adipogenic A medium for 3 days, followed by Adipogenic B medium for 1 day. This cycle was repeated for 21 days of continuous induction. Afterward, the adipogenic differentiation of the cells was observed microscopically. Then, the cells were washed twice with PBS, fixed with 4% paraformaldehyde for 30 min, and rinsed again with PBS. The Oil Red O staining solution (OILR-10001, CYAGEN) was prepared by centrifugation at 1500 rpm for 5 min to remove debris, and the supernatant was used for staining. The solution was added to the cells and incubated at room temperature for 30 min. After three washes with PBS, images of the stained cells were captured using a microscope.

### Alcian blue staining

BMSCs were cultured in a chondrogenic induction medium, with medium replacement every 2–3 days. After 21 days of induction, the chondrogenic differentiation of BMSCs was observed under a microscope. Cells were washed twice with PBS, fixed with 4% paraformaldehyde for 30 min, and washed again with PBS. Alcian Blue staining solution (ALCB-10001, CYAGEN) was applied, and the cells were stained for 30 min while protected from light. After three washes with PBS, the stained cells were imaged under a microscope.

### Extraction and identification of BMSC-EVs

BMSC-EVs were isolated from the BMSC culture supernatant using an exosome extraction kit (EXOQ5A-1, SBI). After culturing BMSCs for 48–72 h in DMEM without serum, the supernatant was collected for EV extraction. The extracted EVs were resuspended in PBS for further analysis. The morphology and size of BMSC-EVs were characterized by transmission electron microscopy (TEM) and nanoparticle tracking analysis (NTA). Protein markers of BMSC-EVs were identified by Western blot using antibodies against CD63 (AWA10080, 1:1000, Abiowell), TSG101 (28283-1-AP, 1:2000, Proteintech), CD9 (20597-1-AP, 1:1000, Proteintech), CD81 (27855-1-AP, 1:1000, Proteintech), and Calnexin (10427-2-AP, 1:5000, Proteintech).

### Phagocytosis experiment

After successful extraction and characterization, BMSC-EVs were stained with PKH67 dye and incubated with Caco-2 cells for treatment. The fluorescence status of the BMSC-EVs within the Caco-2 cells was assessed using a fluorescence microscope.

### Hematoxylin-eosin (HE) staining and Chiu’s score

HE staining was performed on small intestine samples to assess morphological changes. The tissue sections were baked at 60 °C for 1–2 h, dewaxed in water, and stained with hematoxylin for 5–10 min. After rinsing and stained blue in PBS, the sections were counterstained with eosin for 3–5 min. Dehydration was performed using graded ethanol (95–100%) for 5 min at each step, followed by two immersions in xylene for 10 min each. The sections were sealed with neutral gum and analyzed microscopically. Intestinal mucosal injury was graded using Chiu’s scoring criteria [55], with a total score ranging from 0 to 5, and a higher score indicating more severe intestinal mucosal damage.

### Intestinal permeability assay

Serum fluorescein isothiocyanate-dextran (FD-4, Sigma-Aldrich, USA) was used to evaluate intestinal barrier permeability following II/R. Based on a previously established method [56], 200 μL of PBS containing 25 mg/mL FD-4 was administered orally during ischemia. After reperfusion, serum was obtained by centrifugation, and fluorescence intensity was measured using a Varioskan LUX Multimode microplate reader (Thermo Fisher Scientific, USA) at excitation and emission wavelengths of 480 nm and 520 nm, respectively.

### Enzyme-linked immunosorbent assay (ELISA) and biochemical detection

The levels of TNF-α and IL-6 in serum were measured using ELISA kits (TNF-α: KE10002, Proteintech; IL-6: KE10007, Proteintech) according to the manufacturers’ instructions. Additionally, biochemical kits from Nanjing Jiancheng Bioengineering Institute (MDA: A003-1-2, GSH-Px: A005-1-2, GSH: A006-2-1) and Beijing Leagene Biotechnology Co., Ltd. (Fe(II): TC1015) were purchased to determine the levels of MDA, GSH-Px activity, GSH content, and Fe (II) in BMSCs and serum. Blood samples from mice were centrifuged at 1000 × *g* for 15 min at 2–8 °C to separate the serum, which was used immediately for detection. The reagents were equilibrated to room temperature (18–25 °C) for at least 30 min prior to use. Standard solutions, wash buffers, antibody detection working solutions, and horseradish peroxidase (HRP)-labeled avidin working solutions were prepared according to the kit instructions. Standard and sample wells were set up, and 100 μL of either standard solution or sample was added to each well, mixed, and incubated at 37 °C for 2 h. Then, 100 μL of HRP-labeled avidin working solution was added, the plate was sealed and incubated at 37 °C for 1 h. Next, 90 μL of substrate solution was added to each well, and the plate was incubated in the dark at 37 °C for 15–30 min to allow color development. The reaction was terminated by adding 50 μL of stop solution to each well. Optical density (OD) values were measured at 450 nm using a microplate reader within 5 min of stopping the reaction.

For the biochemical detection in BMSCs, the cells were harvested, centrifuged, and the supernatant was discarded. Approximately 5 × 10^7^ cells were resuspended in 1 mL homogenization buffer and centrifuged at 12,000 rpm on ice for 10 min at 4 °C. The supernatant was collected and kept on ice for subsequent analysis. The BCA assay kit (AWB0104, Abiowell) was used for protein quantification following the manufacturer’s instructions. The remaining measurements, including MDA content, GSH-Px activity, GSH content, and Fe(II) content, were conducted according to the respective kit protocols.

### Quantitative real-time PCR (qRT-PCR)

Total RNA was extracted using TRIzol reagent (15596026, Thermo) following the manufacturer’s protocol. RNA was reverse-transcribed into cDNA using the RevertAid First Strand cDNA Synthesis Kit (CW2569, CWBIO). Quantitative PCR was performed on a 7900HT Fast Real-Time PCR System (Applied Biosystems) with the FastStart Universal SYBR Green Master (CW2601, CWBIO). GAPDH and U6 were used as reference genes, and gene expression levels were calculated using the 2^-ΔΔCt^ method. The primer sequences used in the analysis were as follows: GAPDH: Forward (F) ACAGCCTCAAGATCATCAGC, Reverse (R) GGTCATGAGTCCTTCCACGAT; U6: F CTCGCTTCGGCAGCACA, R AACGCTTCACGAATTTGCGT; SREBF2: F CCCTCACCACCCCTATCCAGA, R CTCTTGCCCCATCATTACAGG; HMGB1: F CTATATTACGGTTTGCCCCTT, R ACTGGCACTTTAAGAAAACGAT; miR-182-5p: F ACTGGACTTGGAGTCAGAAGG, R ACTGGACTTGGAGTCAGAAGG; miR-378a-3p: F TTTGGCAATGGTAGAACTCACACCG, R ACTGGACTTGGAGTCAGAAGG; MAPK1: F ATCCCCATCACAAGAAGACCTG, R AGCCTGTTCTACTTCAATCCTCT.

### Western blot

Protein lysates were prepared from cells or tissues using RIPA lysis buffer (AWB0136, Abiowell) supplemented with protease (583794, Gentihold) and phosphatase inhibitors (AWH0650, Abiowell). Proteins were separated by SDS-PAGE and transferred to PVDF membranes. Membranes were blocked with 8% skim milk (AWB0004, Abiowell) for 1 h at room temperature and incubated overnight at 4 °C with the following primary antibodies: SREBF2 (28212-1-AP, 1:1000, Proteintech), HMGB1 (10829-1-AP, 1:500, Proteintech), ACSL4 (66617-1-Ig, 1:3000, Proteintech), GPX4 (67763-1-Ig, 1:1000, Proteintech), FTH1 (#3998, 1:1000, CST), MAPK1 (51068-1-AP, 1:5000, Proteintech), p65 (10745-1-AP, 1:2000, Proteintech), p-p65 (#3033, 1:1000, CST), and β-actin (66009-1-Ig, 1:5000, Proteintech). Horseradish peroxidase-conjugated secondary antibodies were used for detection, and the proteins were visualized using enhanced chemiluminescence (ECL). β-actin was used as a reference to normalize protein expression levels. Full-length Western blots are provided in Supplementary File 1.

### Bioinformatics analysis

Differentially expressed genes were analyzed using the GSE37013 dataset to identify the top 20 upregulated and downregulated genes between the Control and II/R groups, with difference analysis performed using the limma package in R. The cutoff criteria were |logFC| > log_2_(1.5) and *P* < 0.05. The potential binding sites for SREBF2 and HMGB1 were predicted using JASPAR (https://jaspar.genereg.net/analysis). miR-378a-3p binding sites on SREBF2 and MAPK1 were predicted using starBase (https://starbase.sysu.edu.cn/).

### Chromatin immunoprecipitation (Ch-IP)

The enrichment of SREBF2 in the HMGB1 promoter region was assessed using a Ch-IP kit. Briefly, the cells were fixed with 1% formalin for 10 min to crosslink proteins to DNA. DNA fragments of 200–800 bp were generated by sonication, and immunoprecipitation was performed using an antibody specific to SREBF2. The Ch-IP DNA was then purified and eluted in 100 μL of H_2_O. A volume of 2.5 μL of Ch-IPDNA was used for qRT-PCR analysis to assess SREBF2 enrichment at different regions of the HMGB1 promoter. The primer sequences used were as follows: HMGB1-1-F: ACCTGAGGGAACCTAGAAA, HMGB1-1-R: ATTCGGGATTTCAACCACC; HMGB1-2-F: CCAGCATCTTTCTTCTAACA, HMGB1-2-R: CCCTGGGTTTCTCCATTTT; HMGB1-3-F: TAAACCCACCCATTTGAGC, HMGB1-3-R: CCTTTGGTCCTGTTCGTCTC; HMGB1-4-F: ATCATCAACCTTTCCCACTT, HMGB1-4-R: CAGGTAAGGGAGAAGAGGG; HMGB1-5-F: GCGGGCACTCCCCTTCT, HMGB1-5-R: CAGGAAGGAGGACTCTTTTGC; HMGB1-6-F: GGCAAAAGAGTCCTCCTTCCTG, HMGB1-6-R: CCCAAACCCAGCCACTCA; HMGB1-7-F: TTGCGACTGCGGCGACGA, HMGB1-7-R: GTTTTAATCACGCCCCACCC.

### Co-immunoprecipitation (Co-IP)

The interaction between HMGB1 and ACSL4 was assessed using co-IP. The proteins were extracted from the collected cells, and equal amounts of protein extract were incubated overnight at 4 °C in lysis buffer with HMGB1 antibodies for immunoprecipitation. Protein A/G agarose beads were added to the mixture, incubated for 2 h, and washed thrice to remove nonspecific binding. The immunoprecipitated protein complexes were mixed with SDS-PAGE sample buffer, heated, separated by SDS-PAGE, and transferred onto a PVDF membrane. The membrane was probed with antibodies against HMGB1 and ACSL4 and incubated with secondary antibodies. Protein signals were detected using an enhanced chemiluminescence (ECL) reagent and analyzed with an imaging system.

### RNA pull-down

RNA pull-down was performed to validate the interaction between hsa-miR-378a and MAPK1. A biotin-labeled hsa-miR-378a probe was bound to streptavidin magnetic beads. RNA-binding proteins were captured by the probe, and the RNA-protein complexes were washed and eluted. Then, qRT-PCR was performed to detect MAPK1 expression in the eluted complexes. The primer sequences used in the analysis were as follows: MAPK1: F ATCCCCATCACAAGAAGACCTG, MAPK1: R AGCCTGTTCTACTTCAATCCTCT.

### Dual-luciferase reporter assay

The interaction between miR-378a-3p and the MAPK1 or SREBF2 promoter was evaluated using a dual-luciferase reporter assay. Mutant (MUT) and wild-type (WT) human MAPK1 and SREBF2 promoter sequences were synthesized and subcloned into the pGL3-Basic vector by GenePharma (Shanghai, China). The cells were co-transfected with either WT or MUT MAPK1 promoter plasmid (1.5 µg/well) or WT or MUT SREBF2 promoter plasmid (1.5 µg/well) along with mimics NC or miR-378a-3p mimics. TransDetect Double-Luciferase Reporter Assay Kit (FR201, TransGen, China) was used to lyse the transfected cells, and luciferase activity was measured using a Varioskan LUX multimode microplate reader (Thermo Scientific).

### Flow cytometry

ROS levels were analyzed using DCFH-DA. The DCFH-DA dye was diluted 1:1000 to a final concentration of 10 μM and added to cells after removing the cell culture medium, ensuring sufficient coverage of the cells. The cells were incubated with DCFH-DA at 37 °C for 20 min. Unincorporated DCFH-DA was removed by washing the cells three times with a serum-free culture medium. The cells were then collected by trypsinization and analyzed using flow cytometry.

### Transmission electron microscopy (TEM)

The tissues were fixed in 2.5% glutaraldehyde for 6–12 h. After discarding the fixative, the tissues were immersed in PBS buffer for 1–6 h. Then, they were treated with 1% osmium tetroxide for 1–2 h, dehydrated through a graded ethanol series, and macerated. The tissues were embedded in pure epoxy resin and cured in an oven at 40 °C for 12 h, followed by an additional 48 h at 60 °C, which were then sectioned, placed on copper grids, and sequentially stained with lead and uranium solutions. Observations were conducted using a Japan Electron JEM1400 transmission electron microscope.

### Statistical analysis

Statistical analyses were performed using GraphPad Prism 8.0. Measurement data are expressed as mean ± standard deviation. Comparisons between two groups were conducted using the Student’s t-test, while comparisons among multiple groups were analyzed using one-way analysis of variance (ANOVA), followed by Tukey’s post hoc test. The correlation between HMGB1 and SREBP2 expression was assessed using Pearson’s correlation coefficient. A *P* < 0.05 was considered statistically significant.

## Supplementary information

Supplementary Table 1

Supplementary figure legends

Figure S1

Figure S2

Figure S3

Figure S4

Figure S5

Original Data

## Data Availability

All data found and analyzed during this study are included in this paper and its supplementary files. And it is available from the corresponding author or first author upon reasonable request.
