# Supplementary material for: Extracellular vesicles derived from bone marrow mesenchymal stem cells regulate SREBF2/HMGB1 axis by transporting miR-378a-3p to inhibit ferroptosis in intestinal ischemia-reperfusion injury
Source: Cell Death Discov. 2025 May 7;11:223. doi: 10.1038/s41420-025-02509-6 (PMC12058992; doi:10.1038/s41420-025-02509-6)
Supplement: Supplementary file 2 — Supplementary figure legends [file 41420_2025_2509_MOESM2_ESM.docx]

Figure S1. HMGB1 and SREBF2 expressions and the flowchart of the animal handling process. A. Top 20 differentially up- and down-regulated genes in Control and II/R groups. B. HMGB1 and SREBF2 expressions in Control and II/R groups. * P<0.05 vs Control. C. qRT-PCR was used to detect the expression of SREBF2 and HMGB1. * P<0.05 vs Sham. D. The flowchart of the animal handling process.

Figure S2. The efficiency of knockdown of HMGB1 and the correlation of HMGB1 and SREBF2. A. The efficiency of knockdown of HMGB1 in Caco-2 cells. * P<0.05 vs sh-NC. B. The correlation between HMGB1 and SREBF2 was analyzed using Pearson correlation coefficients. C. Western blot analysis of SREBF2 expression in H/R-induced Caco-2 cells. * P<0.05 vs Control. D. JASPAR (https://jaspar.genereg.net/analysis) prediction of the binding sites for SREBF2 and HMGB1. E. The efficiency of knockdown of SREBF2 in Caco-2 cells. * P<0.05 vs sh-NC.

Figure S3. Extraction and identification of BMSC-EVs. A-C. The osteogenic differentiation, adipogenic differentiation, and chondrogenic differentiation of BMSCs. D. TEM for identification of BMSC-EVs. scale bar = 500 nm. E. Diameter analysis of BMSC-EVs. F. Western blot analysis of CD63, TSG101, CD81, CD9, and Calnexin expressions in BMSC-EVs. G. Phagocytosis experiment in Caco-2 cells. scale bar = 25 μm (400×).

Figure S4. Validation of the targeting relationship between miR-378a-3p and SREBF2. A. Bioinformatics prediction of the targeted binding sites of miR-378a-3p to SREBF2. B. Dual-luciferase reporter assay verification of miR-378a-3p binding to SREBF2. ns, no significance.

Figure S5. qRT-PCR detection of miR-378a-3p expression. * P<0.05 vs BMSC.
