# Supplementary figures and images for: Extracellular vesicles derived from bone marrow mesenchymal stem cells regulate SREBF2/HMGB1 axis by transporting miR-378a-3p to inhibit ferroptosis in intestinal ischemia-reperfusion injury
Source: Cell Death Discov. 2025 May 7;11:223. doi: 10.1038/s41420-025-02509-6 (PMC12058992; doi:10.1038/s41420-025-02509-6)

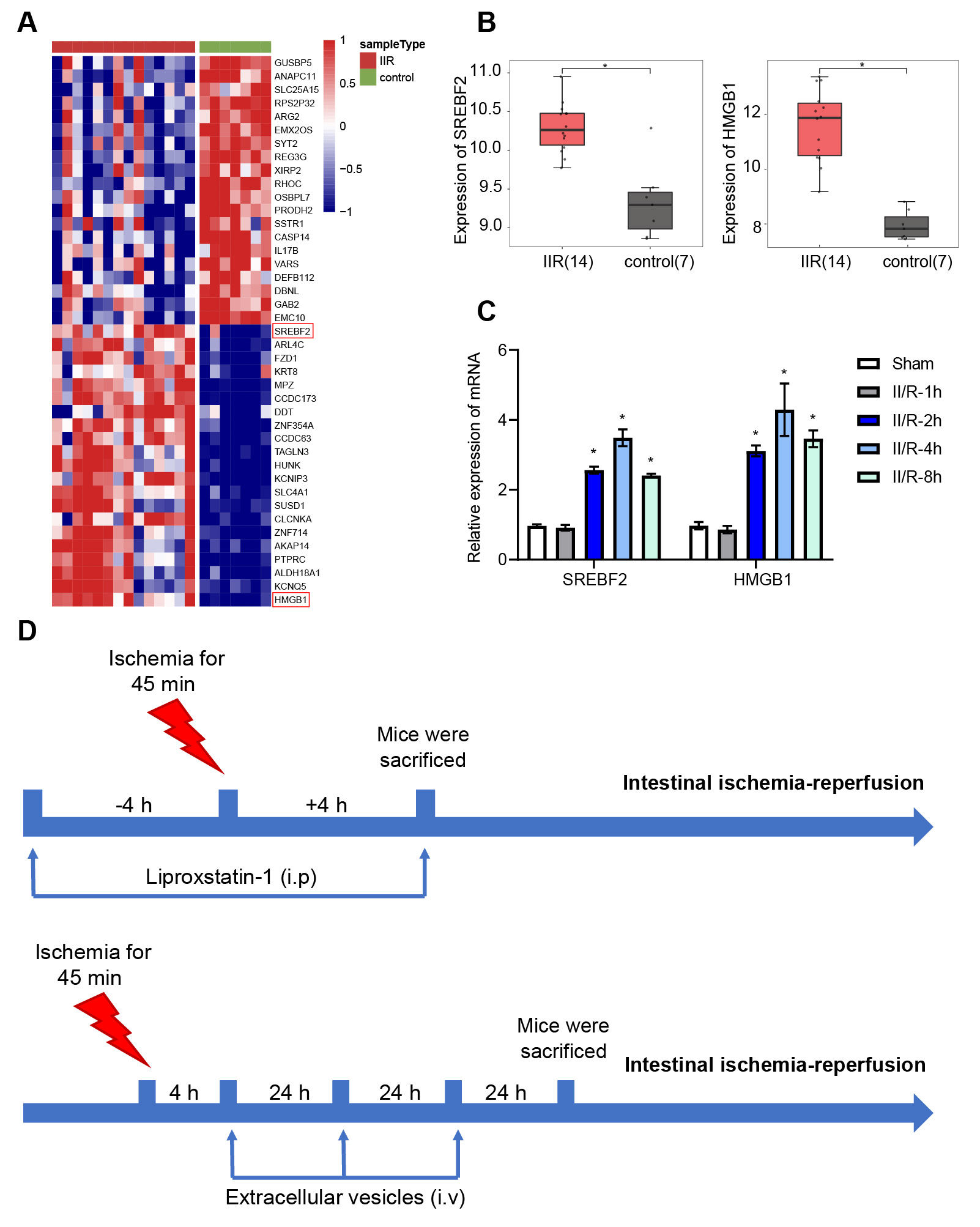

Supplement: Supplementary file 3 — Figure S1 [file 41420_2025_2509_MOESM3_ESM.jpg]

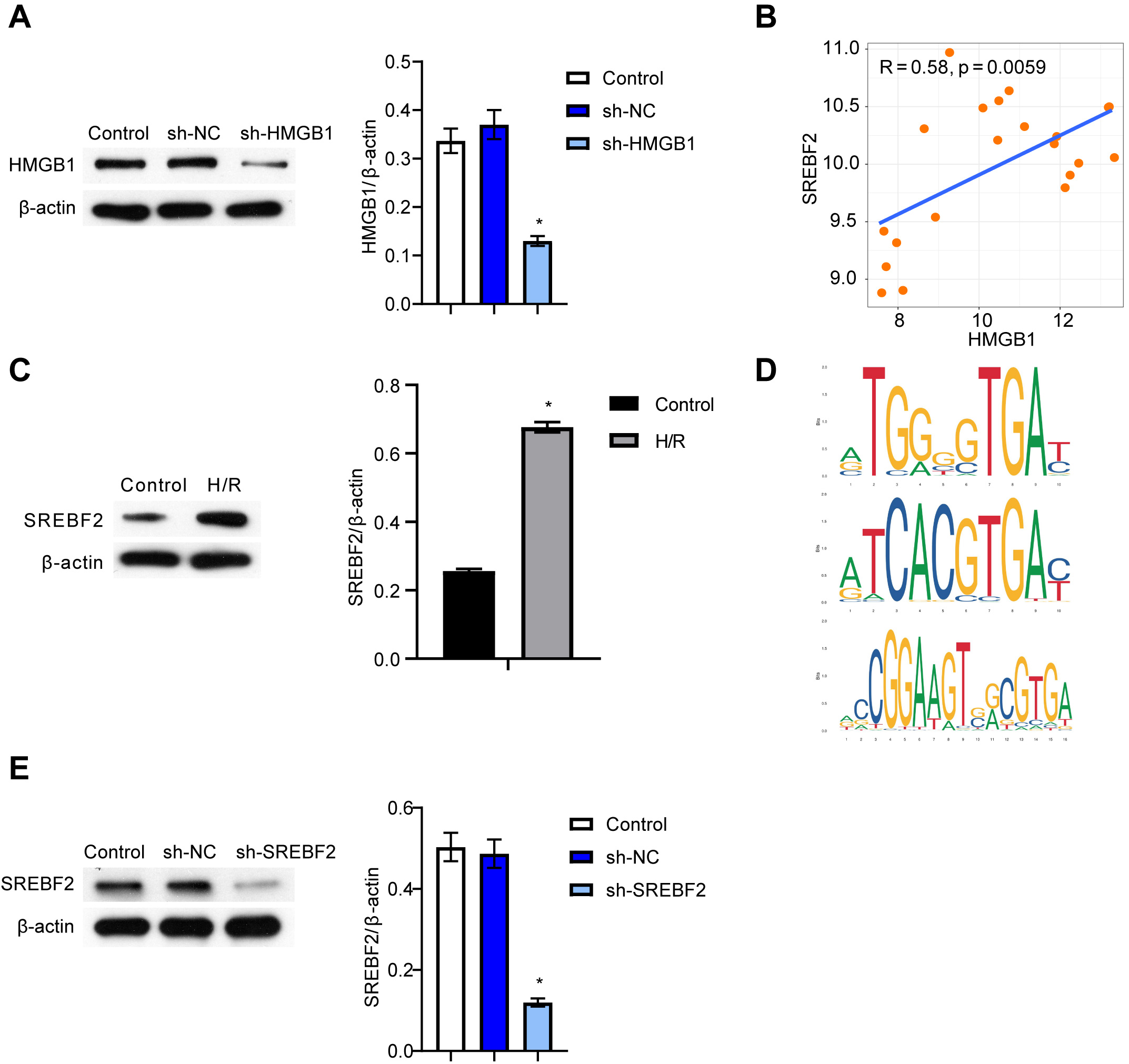

Supplement: Supplementary file 4 — Figure S2 [file 41420_2025_2509_MOESM4_ESM.jpg]

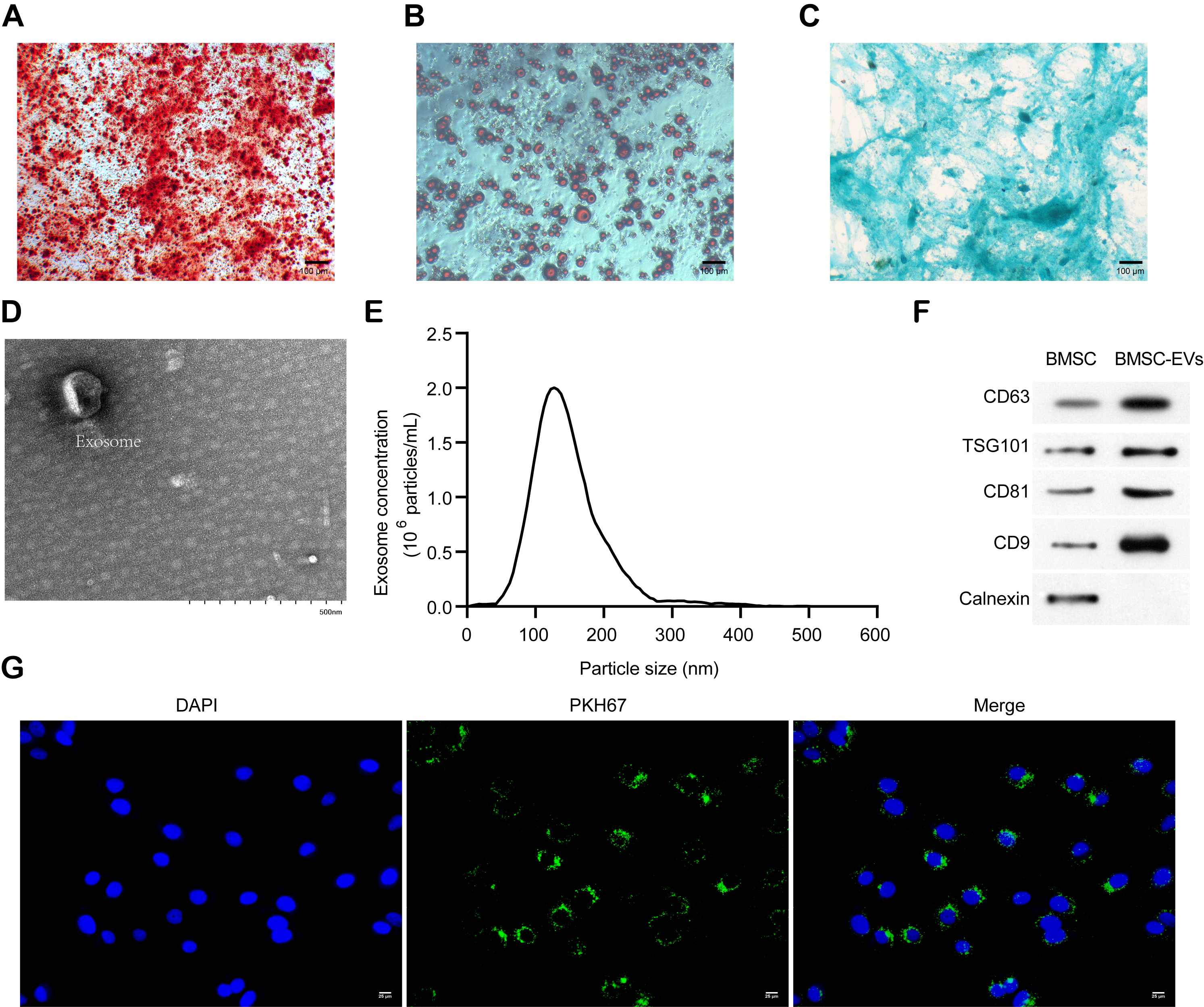

Supplement: Supplementary file 5 — Figure S3 [file 41420_2025_2509_MOESM5_ESM.jpg]

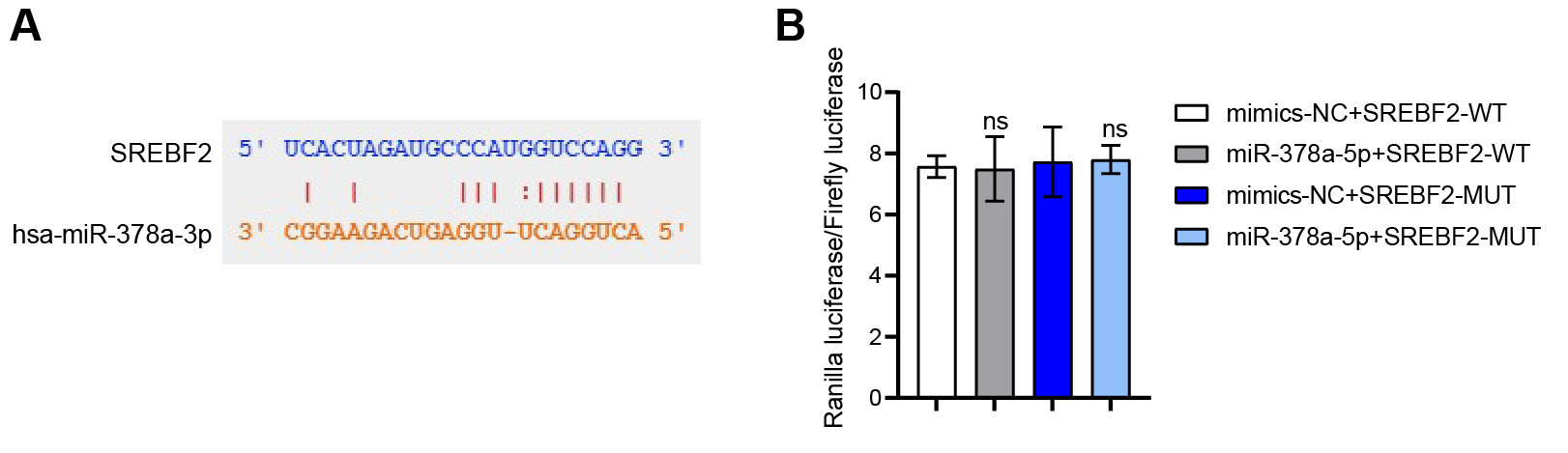

Supplement: Supplementary file 6 — Figure S4 [file 41420_2025_2509_MOESM6_ESM.jpg]

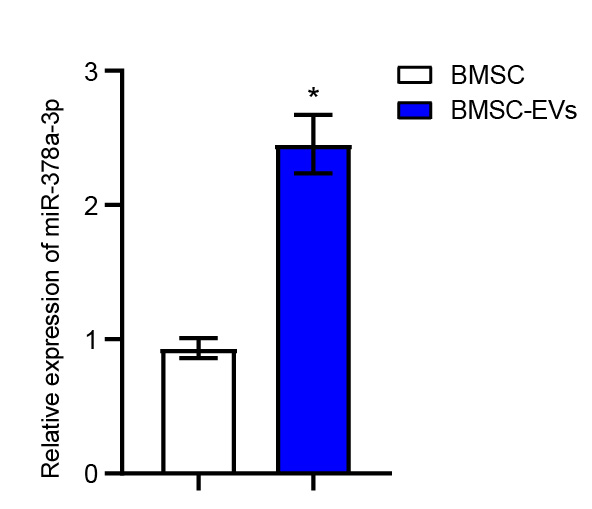

Supplement: Supplementary file 7 — Figure S5 [file 41420_2025_2509_MOESM7_ESM.jpg]
