# Supplementary material for: Extracellular vesicles derived from bone marrow mesenchymal stem cells regulate SREBF2/HMGB1 axis by transporting miR-378a-3p to inhibit ferroptosis in intestinal ischemia-reperfusion injury
Source: Cell Death Discov. 2025 May 7;11:223. doi: 10.1038/s41420-025-02509-6 (PMC12058992; doi:10.1038/s41420-025-02509-6)
Supplement: Supplementary file 8 — Original Data [file 41420_2025_2509_MOESM8_ESM.pdf]

Figure 1. Lip or HMGB1 inhibited ferroptosis induced by II/R. For animal experiments, 10 mg/kg Lip was injected intraperitoneally 4 hours before and after ischemia to evaluate its effect on II/R-induced ferroptosis 4,20. For cell experiments, Caco-2 cells were treated with Lip at a final concentration of 200 nM for 12 hours before inducing hypoxia. A. HE staining illustrating intestinal tissue damage and Chiu's histological score for intestinal injury. Scale bar = 100 μm (100×), 25 μm (400×). B. Intestinal permeability was determined using fluorescein isothiocyanate-dextran (FD-4). C. ELISA analysis of TNF-α and IL-6 levels. D. ROS levels were assessed by flow cytometry. E and F. Western blot analysis of GPX4 and HMGB1 expression. G. TEM analysis of ferroptosis. Red arrows indicate ferroptotic mitochondria, and yellow arrows indicate normal mitochondria. Scale bar = 500 nm. H. Western blot detection of HMGB1 expression in intestinal tissue. I. HE staining and Chiu's histological score for intestinal injury. Scale bar = 100 μm (100×), 25 μm (400×). J. Intestinal permeability assessment. K. ELISA detection of inflammatory cytokines TNF-α and IL-6. L. Lipid ROS levels in tissues were analyzed by flow cytometry. M. Western blot analysis of GPX4, HMGB1, and ACSL4 expression in tissues. * $P<0.05$ vs Sham or Control, # $P<0.05$ vs II/R or H/R. For animal experiments, n=6 mice per group. For Western blot experiments, n=3.

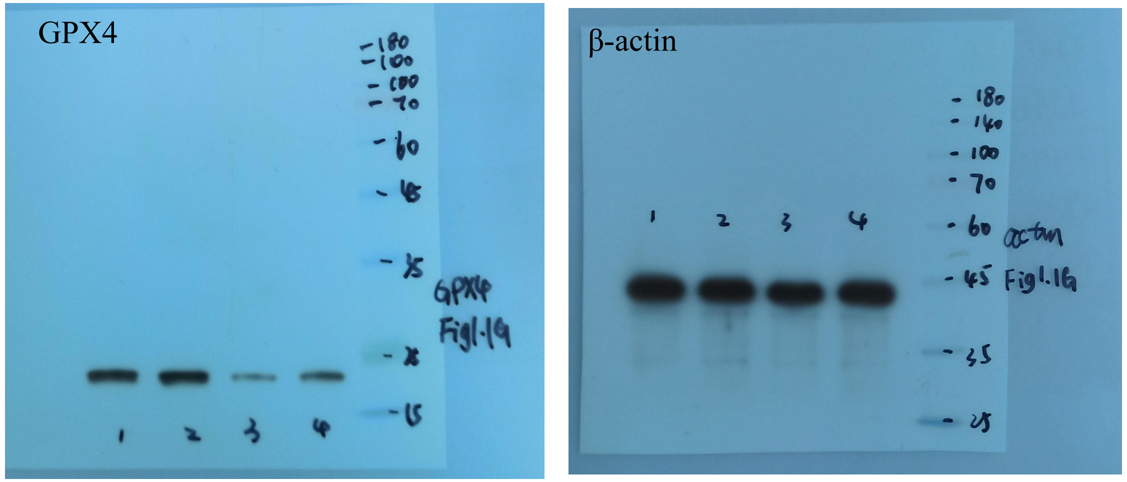

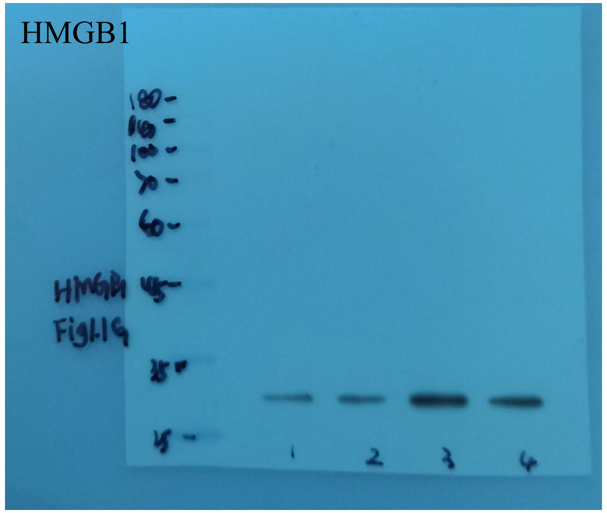

Fig. 1E
1 Sham
2 Sham+Lip
3 II/R
4 II/R+Lip

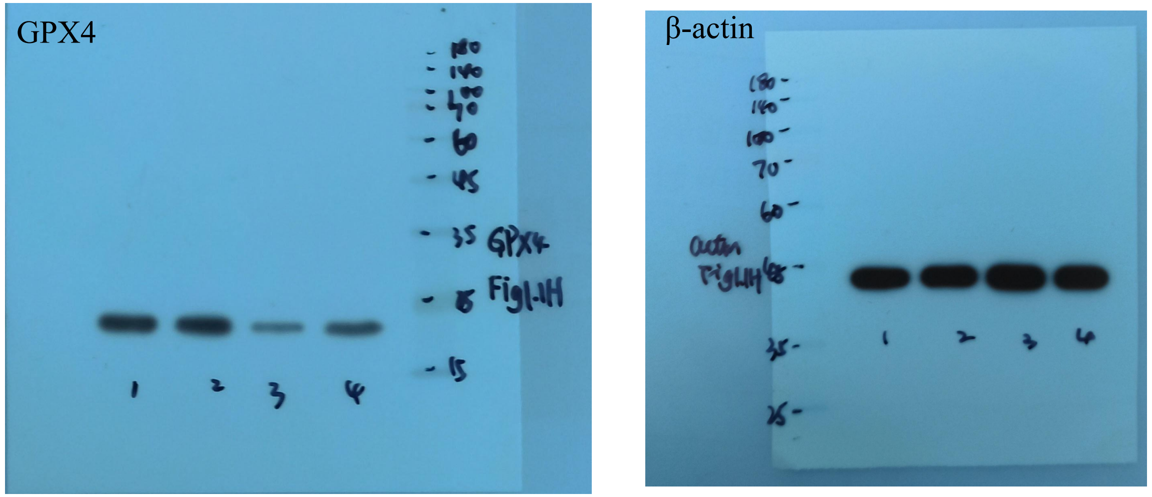

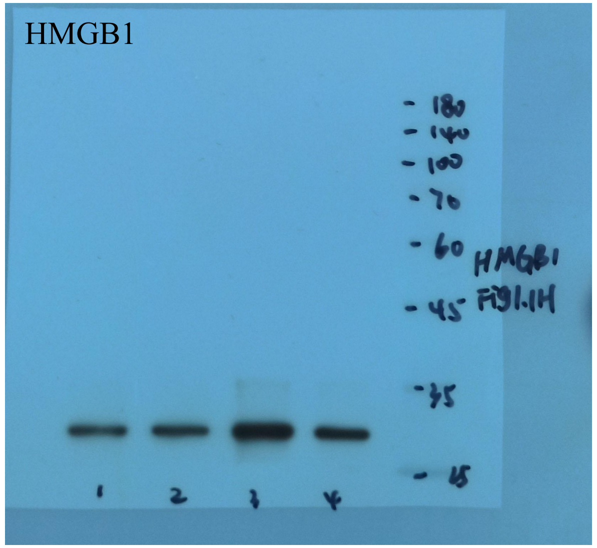

Fig. 1F
1 Control
2 Control +Lip
3 H/R
4 H/R+Lip

Figure 1. Lip or HMGB1 inhibited ferroptosis induced by II/R. For animal experiments, 10 mg/kg Lip was injected intraperitoneally 4 hours before and after ischemia to evaluate its effect on II/R-induced ferroptosis 4,20. For cell experiments, Caco-2 cells were treated with Lip at a final concentration of 200 nM for 12 hours before inducing hypoxia. A. HE staining illustrating intestinal tissue damage and Chiu's histological score for intestinal injury. Scale bar = 100 μm (100×), 25 μm (400×). B. Intestinal permeability was determined using fluorescein isothiocyanate-dextran (FD-4). C. ELISA analysis of TNF-α and IL-6 levels. D. ROS levels were assessed by flow cytometry. E and F. Western blot analysis of GPX4 and HMGB1 expression. G. TEM analysis of ferroptosis. Red arrows indicate ferroptotic mitochondria, and yellow arrows indicate normal mitochondria. Scale bar = 500 nm. H. Western blot detection of HMGB1 expression in intestinal tissue. I. HE staining and Chiu's histological score for intestinal injury. Scale bar = 100 μm (100×), 25 μm (400×). J. Intestinal permeability assessment. K. ELISA detection of inflammatory cytokines TNF-α and IL-6. L. Lipid ROS levels in tissues were analyzed by flow cytometry. M. Western blot analysis of GPX4, HMGB1, and ACSL4 expression in tissues. * P<0.05 vs Sham or Control, # P<0.05 vs II/R or H/R. For animal experiments, n=6 mice per group. For Western blot experiments, n=3.

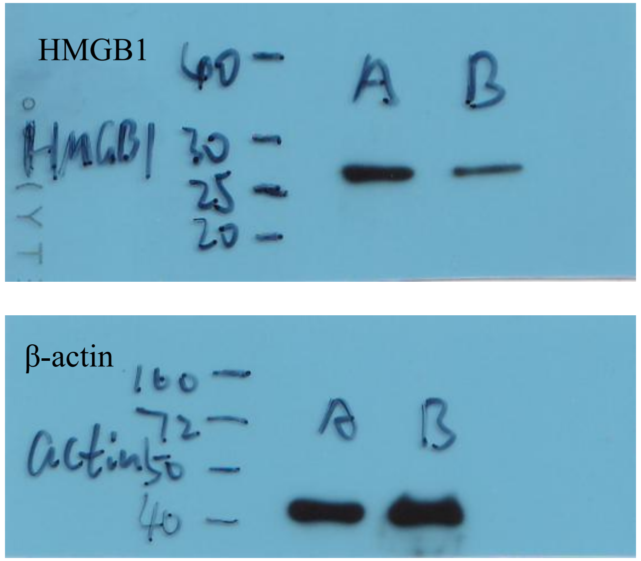

Fig. 1H
1 II/R+sh-NC
2 II/R+sh-HMGB1

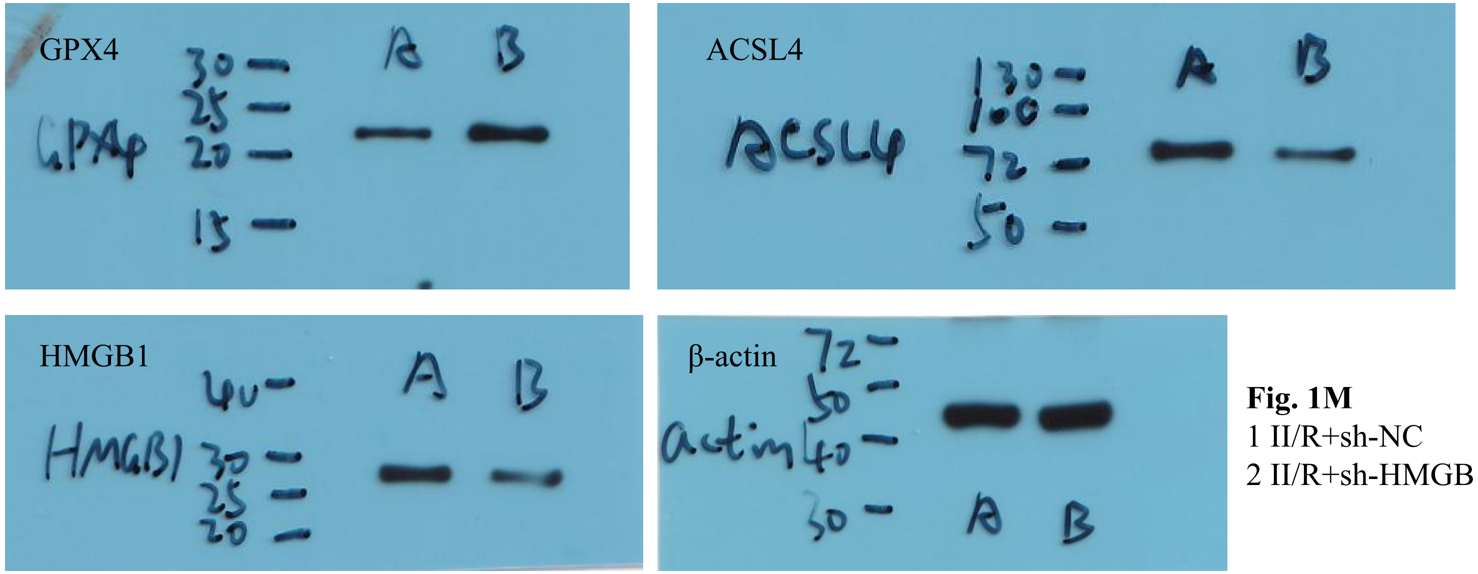

Fig. 1M
1 II/R+sh-NC
2 II/R+sh-HMGB1

Figure 2. Knockdown of HMGB1 inhibited ferroptosis induced by H/R in Caco-2 cells. A. Western blot analysis of HMGB1 expression. B. Biochemical assays measuring MDA content, GSH-Px activity, GSH content, and Fe (II) levels. C. Western blot detection of ferroptosis-related protein levels (ACSL4, GPX4, and FTH1). D. ROS levels assessed by flow cytometry. E. Co-IP analysis demonstrating the interaction between HMGB1 and ACSL4. For cell experiments, n=3. For Western blot experiments, n=3. * P<0.05 vs H/R, # P<0.05 vs H/R+sh-NC.

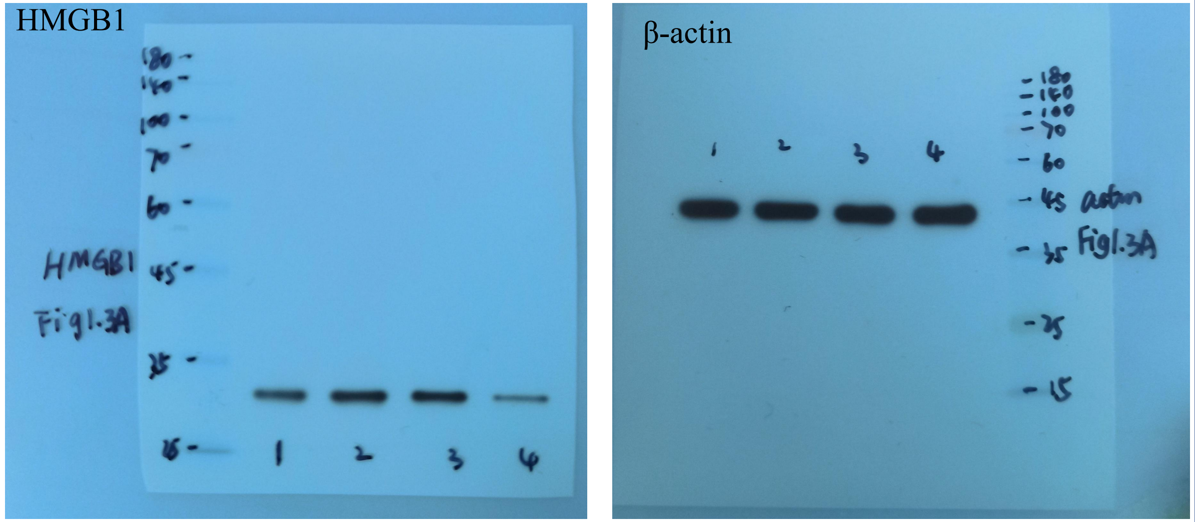

Fig. 2A

1 Control 2 H/R 3 H/R+sh-NC 4 H/R+sh-HMGB1

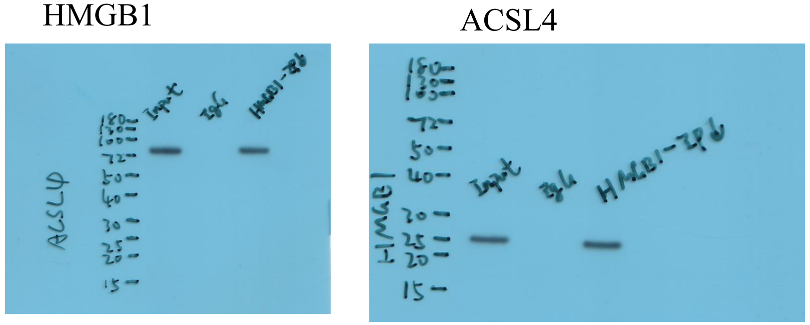

Fig. 2E

1 Input 2 IgG 3 HMGB1-IP

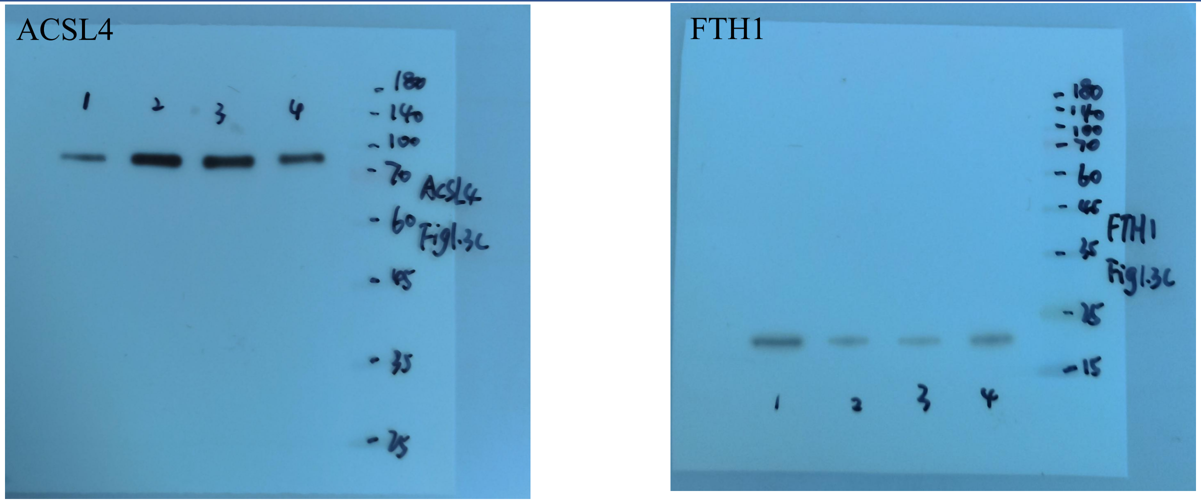

Fig. 2C

1. Control 2. H/R 3. H/R+sh-NC 4. H/R+sh-HMGB1

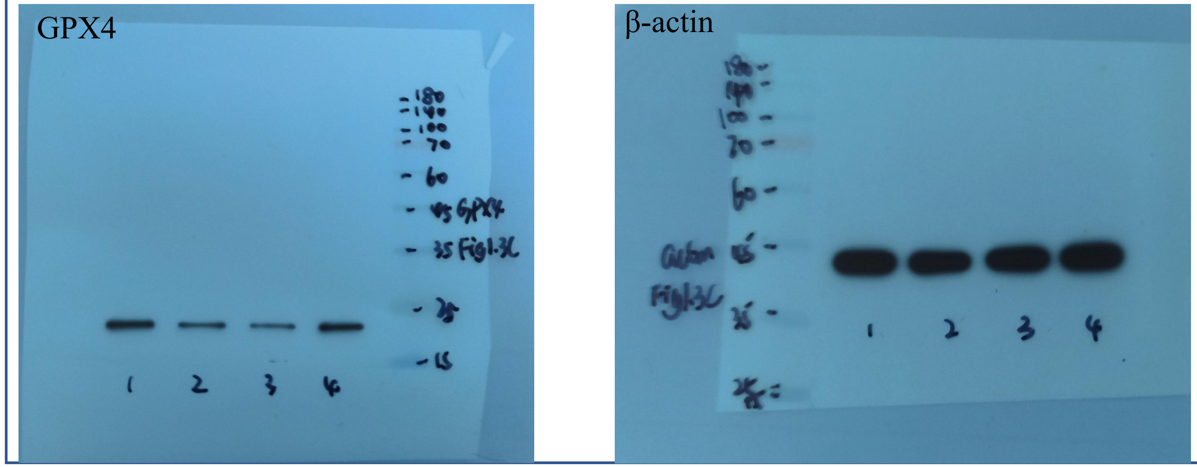

Figure 3. SREBF2 directly regulated HMGB1 expression to promote H/R-induced ferroptosis in Caco-2 cells. A. Ch-IP analysis verified the binding of SREBF2 to the HMGB1 promoter (*P<0.05 vs IP). B. Western blot detection of SREBF2 and HMGB1 expression levels. C. Biochemical assays measuring MDA content, GSH-Px activity, GSH content, and Fe (II) levels. D. Western blot analysis of ferroptosis-related proteins (ACSL4, GPX4, and FTH1). E. Flow cytometry analysis of ROS levels. For cell experiments, n=3. For Western blot experiments, n=3. * P<0.05 vs H/R+sh-NC.

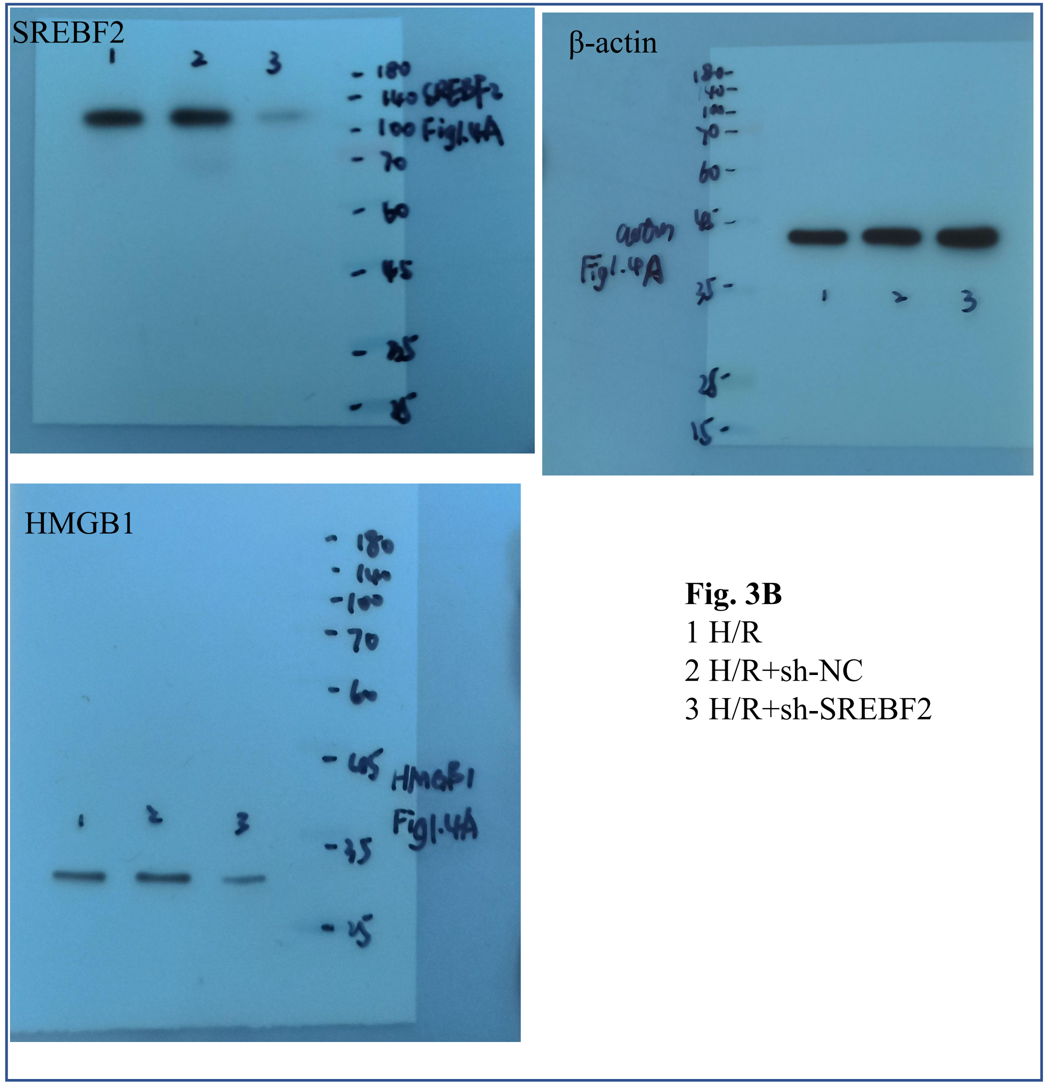

Fig. 3B
1 H/R
2 H/R+sh-NC
3 H/R+sh-SREBF2

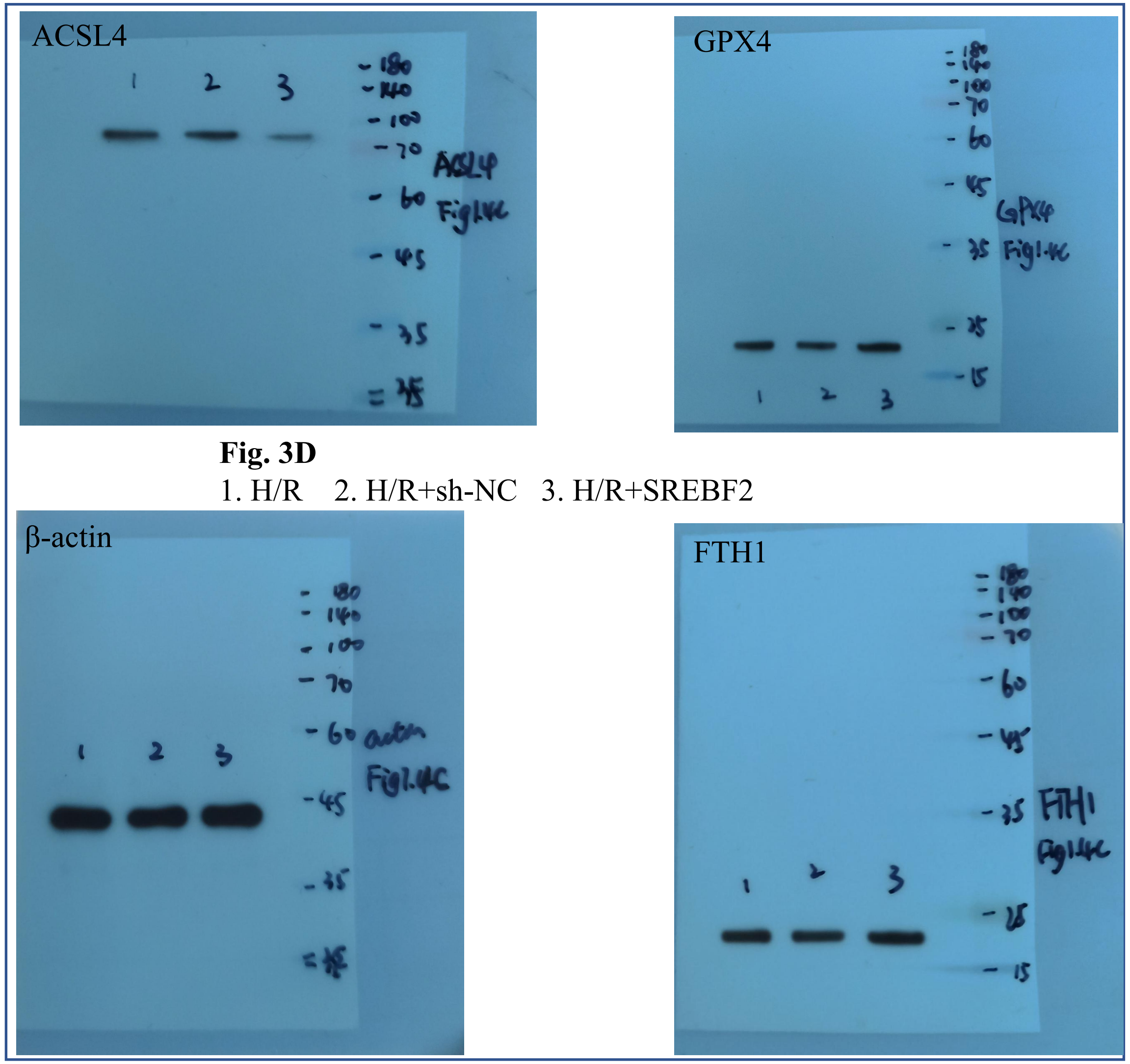

Fig. 3D
1. H/R 2. H/R+sh-NC 3. H/R+SREBF2

Figure 4. BMSC-EVs suppressed SREBF2/HMGB1 axis and alleviated H/R-induced ferroptosis in Caco-2 cells. A. Biochemical assays measuring MDA content, GSH-Px activity, GSH content, and Fe (II) levels. B. ROS levels analyzed by flow cytometry. C. Western blot detection of ferroptosis-related proteins (ACSL4, GPX4, FTH1, SREBF2, and HMGB1). For cell experiments, n=3. For Western blot experiments, n=3. * P<0.05 vs Control, #P<0.05 vs H/R.

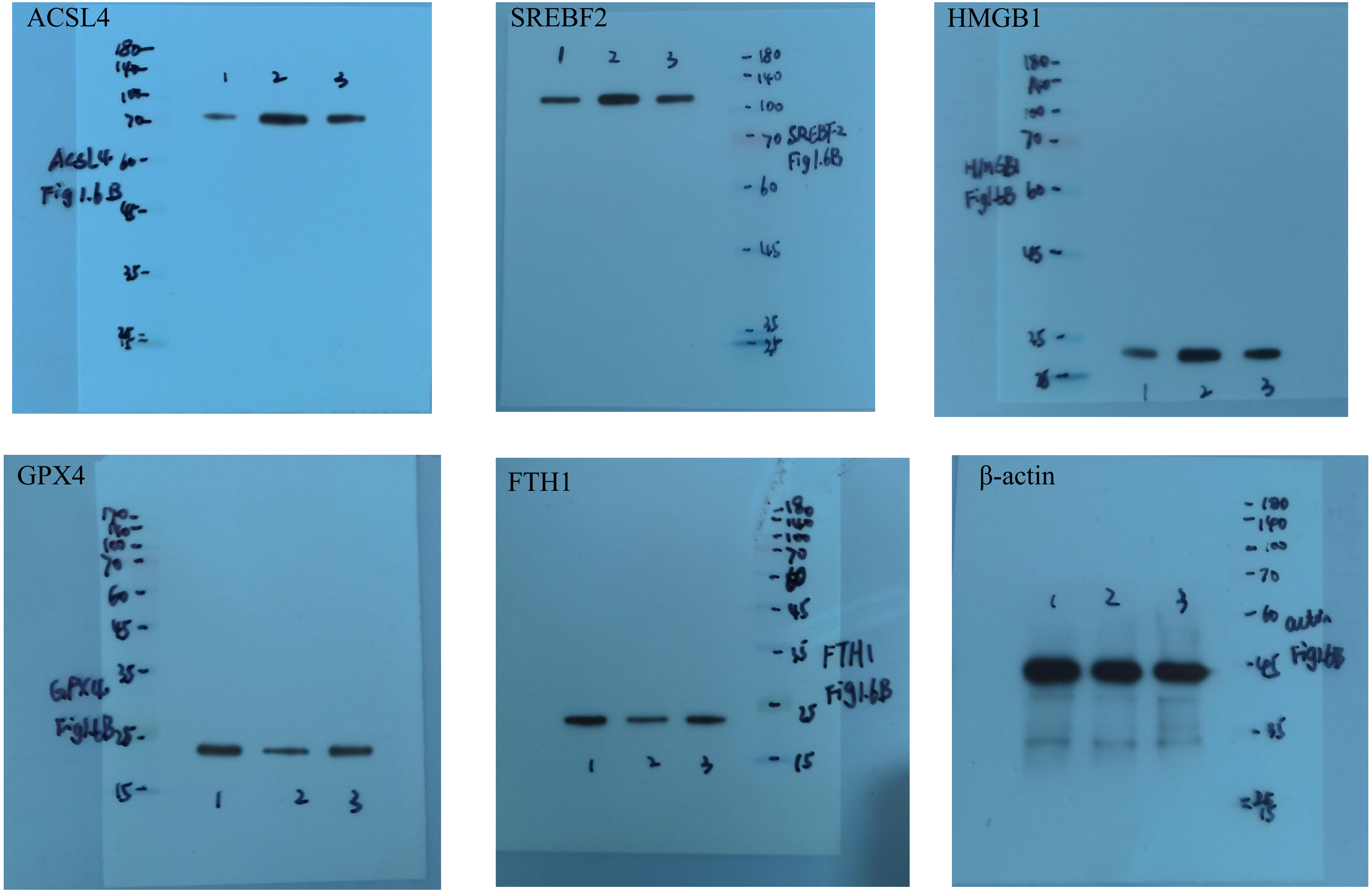

Fig. 4C
1 Control
2 H/R
3 Evs

Figure 5. miR-378a-3p regulated the SREBF2/HMGB1 axis. A-D. qRT-PCR analysis of miR-182-5p and miR-378a-3p expression. E. qRT-PCR assessment of SREBF2 and HMGB1 expression levels. F and G. qRT-PCR analysis of miR-378a-3p, SREBF2 and HMGB1 expression levels. H. Bioinformatics prediction of miR-378a-3p binding sites on MAPK1. I. Dual-luciferase reporter assay confirming the interaction between hsa-miR-378a-3p and MAPK1. J. RNA pull-down assay validating the interaction between hsa-miR-378a-3p and MAPK1. K. qRT-PCR detection of MAPK1 expression. L. Western blot analysis of MAPK1, p65, and p-p65 expression. For animal experiments, n=6 mice per group. For cell experiments, n=3. For Western blot experiments, n=3. * P<0.05 vs Sham, Control, mimics NC, inhibitor NC or Bio-miR-378a-NC.

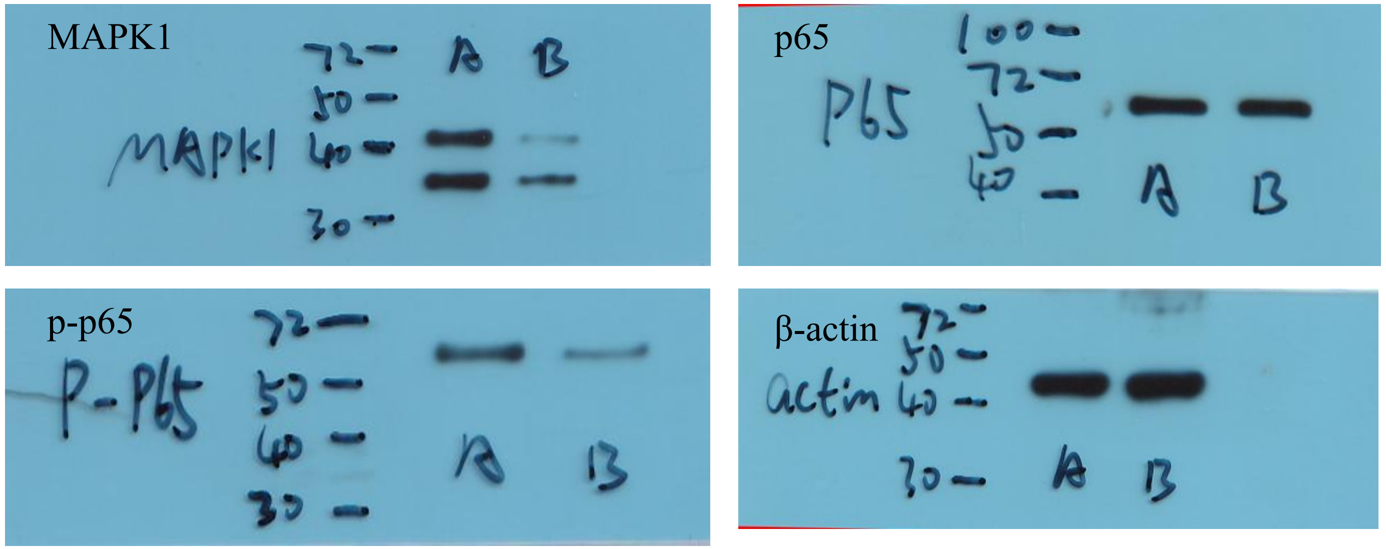

Fig. 5L
1 mimics NC
2 miR-378a-3p mimics

Figure 6. miR-378a-3p derived from BMSC-EVs regulated SREBF2/HMGB1 axis to inhibit H/R-induced ferroptosis in Caco-2 cells. A. miR-378a-3p expression determined by qRT-PCR (*P<0.05 vs EVs-inhibitor NC). B. qRT-PCR analysis of miR-378a-3p expression (*P<0.05 vs EVs-mimics NC). C. qRT-PCR detection of miR-378a-3p expression (*P<0.05 vs H/R, #P<0.05 vs H/R+EVs-NC). D. Biochemical analysis of MDA content, GSH-Px activity, GSH content, and Fe (II) levels. E. Western blot detection of ferroptosis-related proteins ACSL4, GPX4, FTH1, SREBF2, and HMGB1, along with MAPK1, p65, and p-p65 levels. F. ROS levels measured by flow cytometry. For cell experiments, n=3. For Western blot experiments, n=3. * P<0.05 vs H/R, #P<0.05 vs H/R+EVs-inhibitor NC.

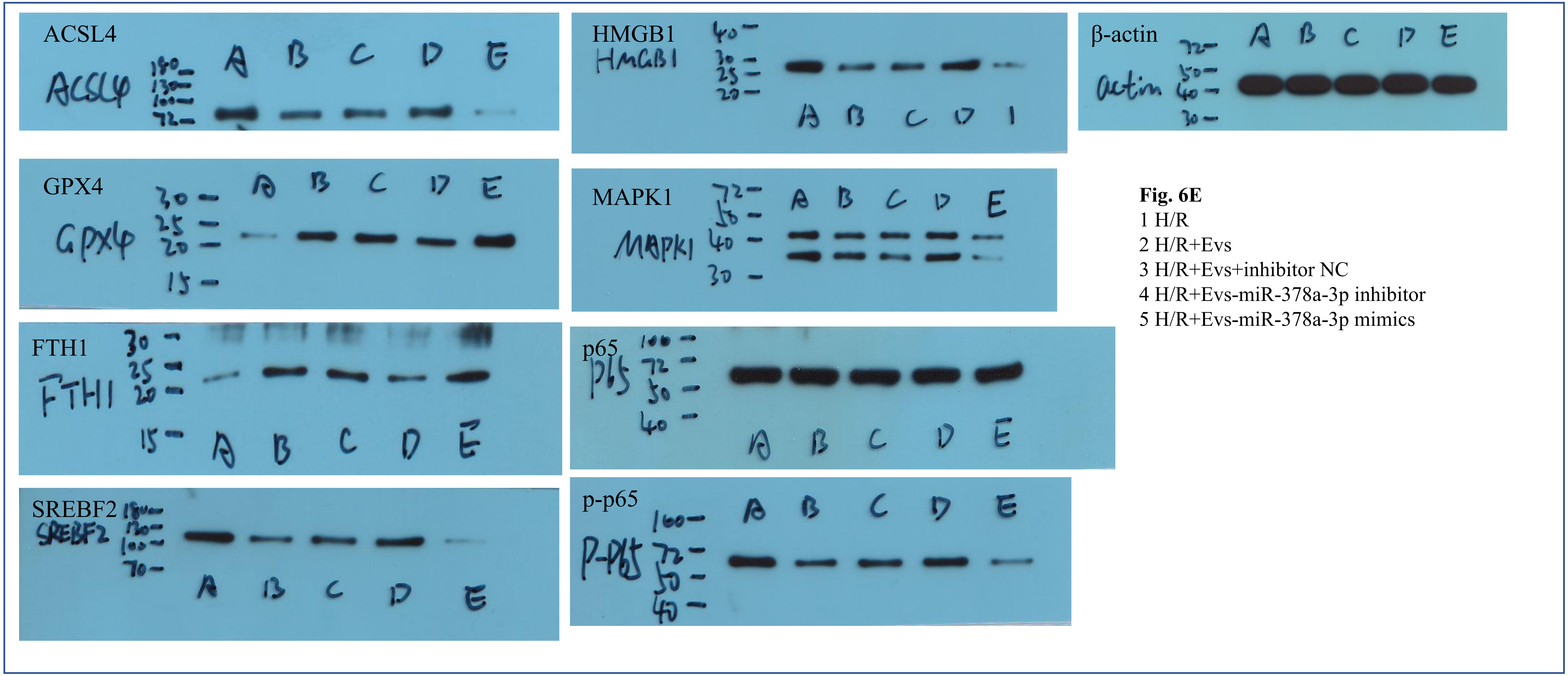

Figure 7. miR-378a-3p derived from BMSC-EVs alleviated II/R-induced ferroptosis via SREBF2/HMGB1 axis. A. HE staining of intestinal tissue damage and Chiu's histological score for intestinal injury. Scale bar = 100 μm (100×), 25 μm (400×). B. miR-378a-3p expression monitored through qRT-PCR. C. Biochemical analysis of MDA content, GSH-Px activity, GSH content, and Fe (II) levels. D. Western blot detection of ferroptosis-related proteins ACSL4, GPX4, FTH1, SREBF2, HMGB1, MAPK1, p65 and p-p65 levels. E. ROS levels measured by flow cytometry. F. TEM analysis of ferroptosis. Red arrows indicate ferroptotic mitochondria, and yellow arrows indicate normal mitochondria. Scale bar = 500 nm. For animal experiments, n=3. For Western blot experiments, n=3. * $P<0.05$ vs II/R, #$P<0.05$ vs EVs-mimics NC.

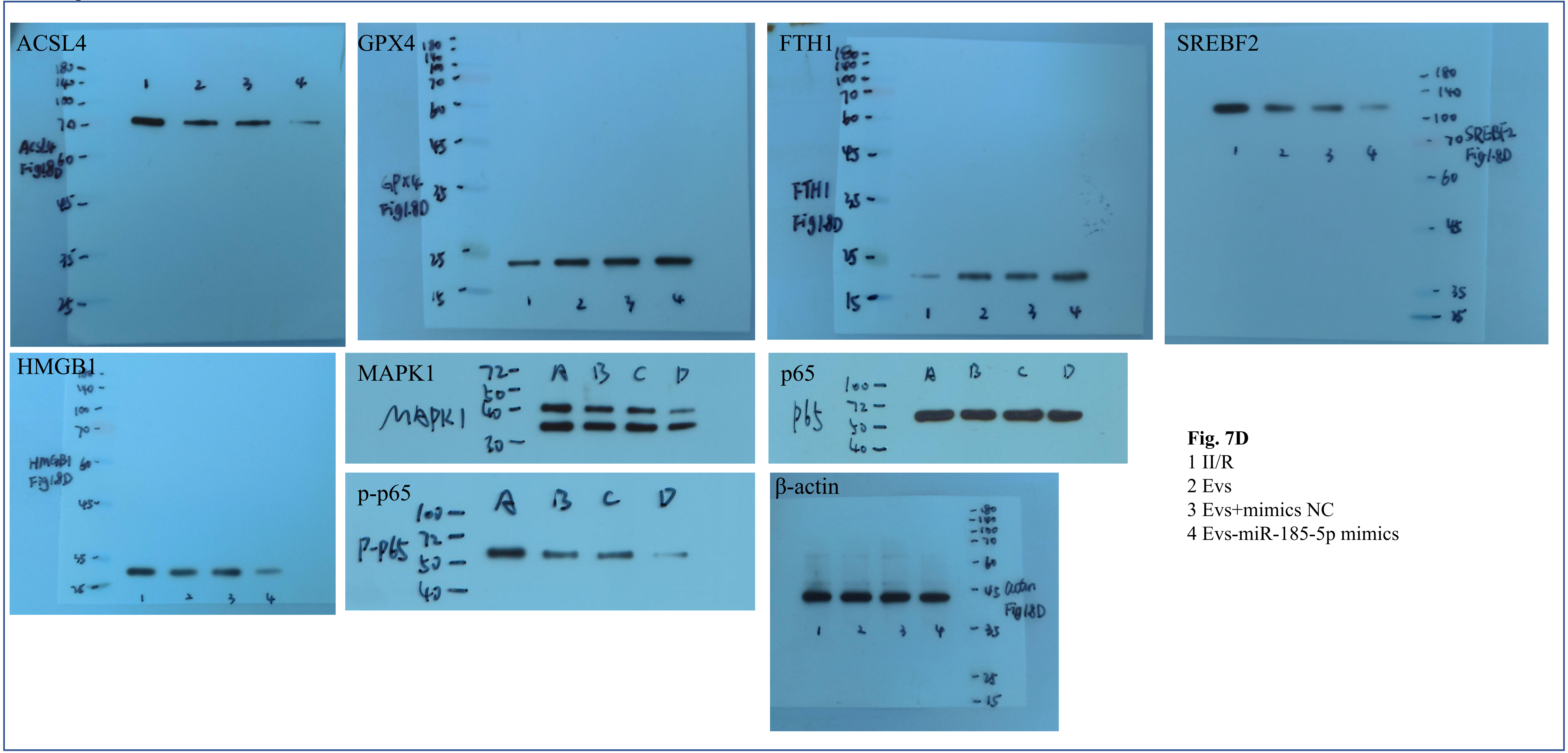

Figure S2. The efficiency of knockdown of HMGB1 and the correlation of HMGB1 and SREBF2. A. The efficiency of knockdown of HMGB1 in Caco-2 cells. * P<0.05 vs sh-NC. B. The correlation between HMGB1 and SREBF2 was analyzed using Pearson correlation coefficients. C. Western blot analysis of SREBF2 expression in H/R-induced Caco-2 cells. * P<0.05 vs Control. D. JASPAR (https://jaspar.genereg.net/analysis) prediction of the binding sites for SREBF2 and HMGB1. E and F. The efficiency of knockdown and overexpression of SREBF2 in Caco-2 cells. * P<0.05 vs sh-NC or oe-NC.

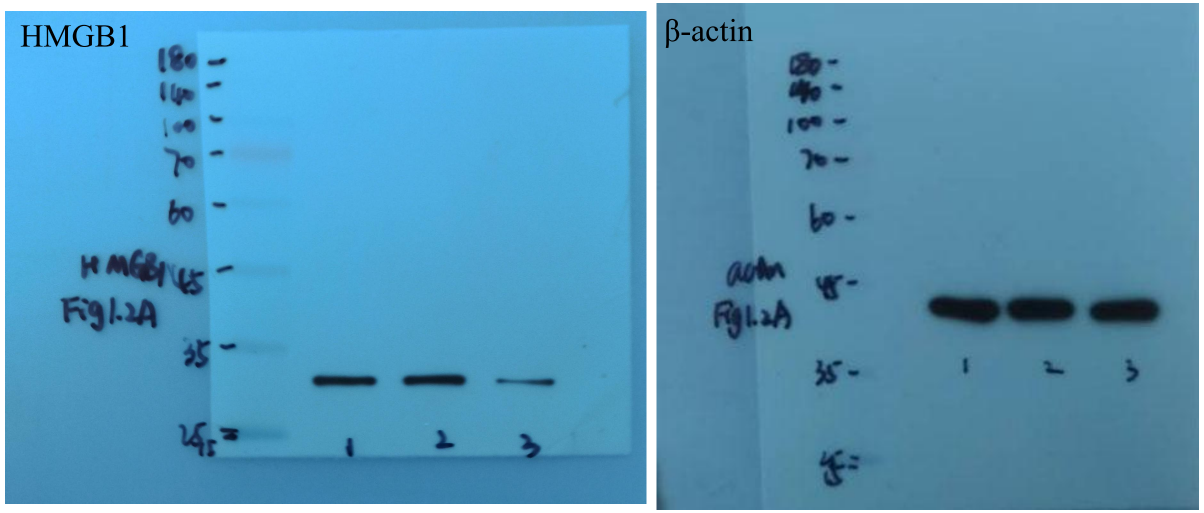

Fig. S2A

1 Control
2 sh-NC
3 sh-HMGB1

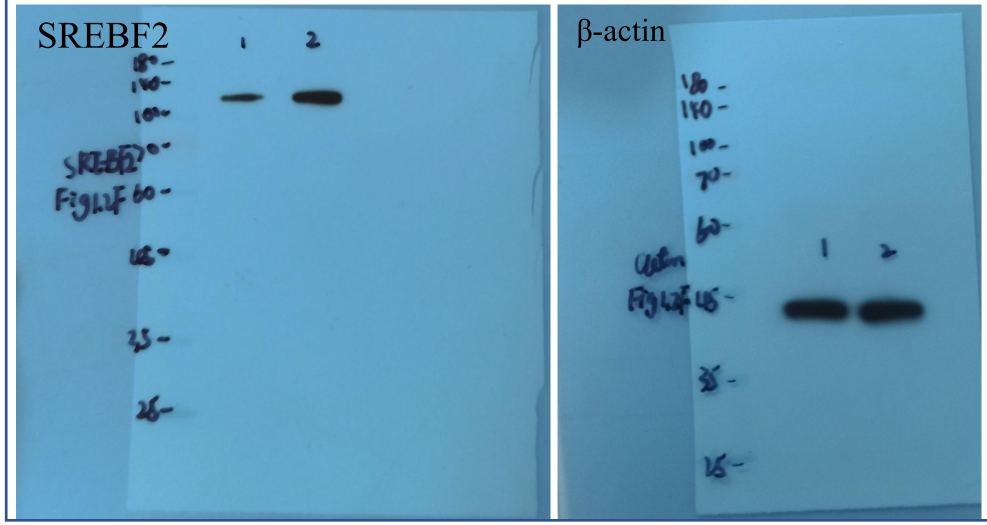

Fig. S2B
1 Control
2 H/R

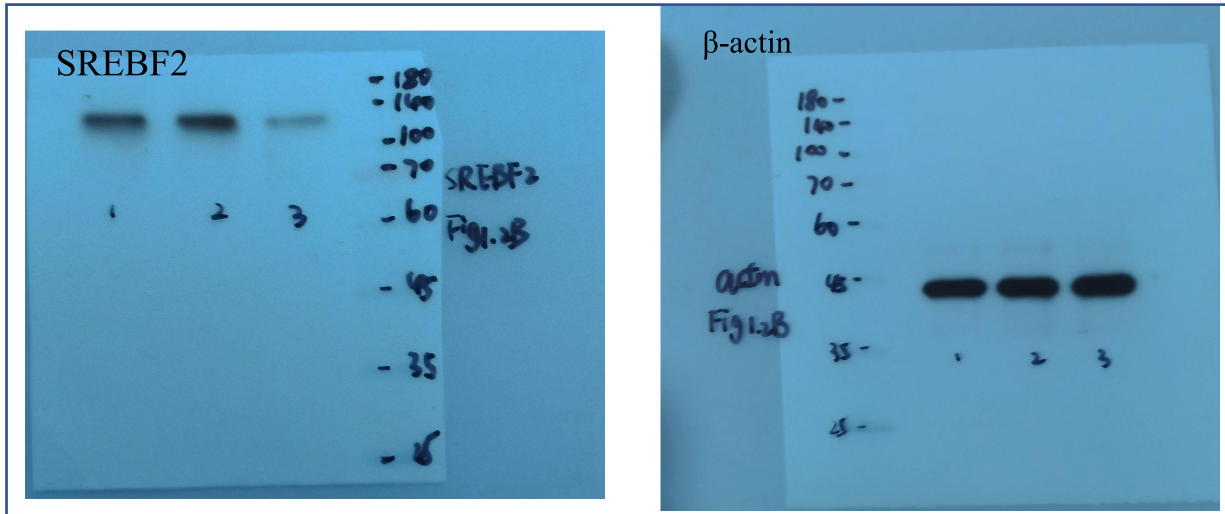

Fig. S2E 1 Control
2 sh-NC
3 sh-SREBF2

Figure S3. Extraction and identification of BMSC-EVs. A-C. The osteogenic differentiation, adipogenic differentiation, and chondrogenic differentiation of BMSCs. D. TEM for identification of BMSC-EVs. scale bar = 500 nm. E. Diameter analysis of BMSC-EVs. F. Western blot analysis of CD63, TSG101, CD81, CD9, and Calnexin expressions in BMSC-EVs. G. Phagocytosis experiment in Caco-2 cells. scale bar = 25 μm (400×).

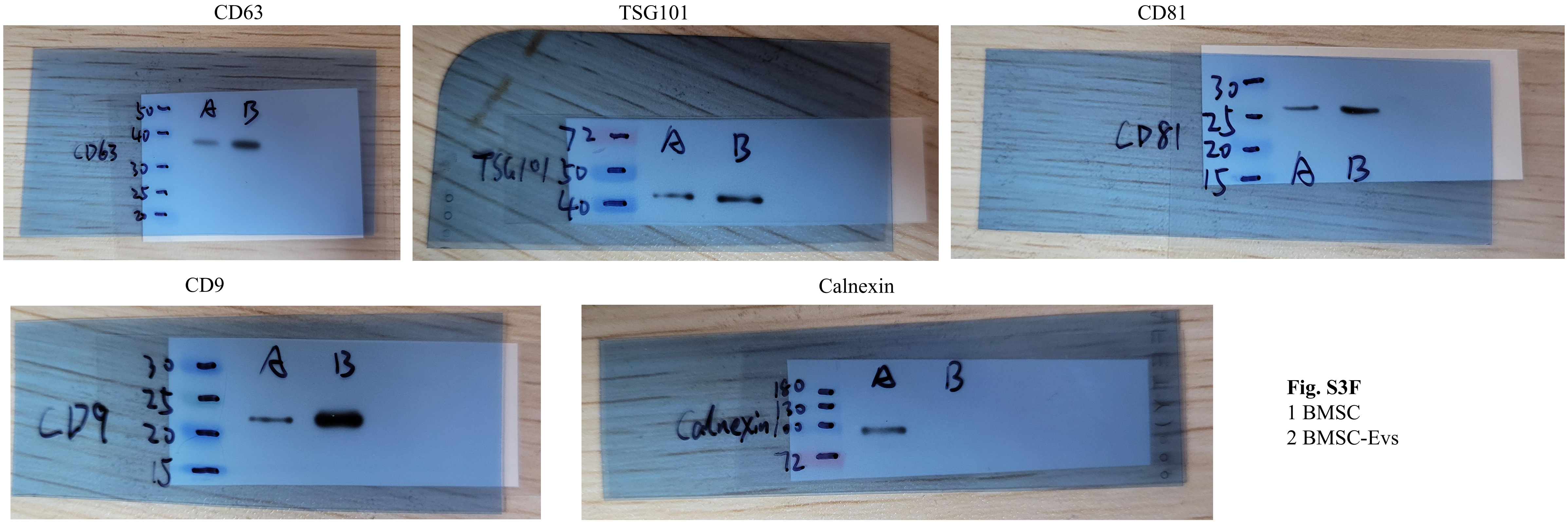

Fig. S3F
1 BMSC
2 BMSC-Evs
